# Effect of Cell Cycle on Cell Surface Expression of Voltage-Gated Sodium Channels and Na^+^,K^+^-ATPase

**DOI:** 10.3390/cells11203240

**Published:** 2022-10-15

**Authors:** Samantha Edenfield, Abigail M. Sims, Constance Porretta, Harry J. Gould, Dennis Paul

**Affiliations:** 1 Department of Pharmacology and Experimental Therapeutics, Louisiana State University Health Sciences Center, New Orleans, LA 70112, USA; 2 Department of Microbiology and Immunology, Tulane University School of Medicine, New Orleans, LA 70112, USA; 3 Department of Neurology, Louisiana State University Health Sciences Center, New Orleans, LA 70112, USA; 4 Department of Anesthesiology, Louisiana State University Health Sciences Center, New Orleans, LA 70112, USA; 5 Neuroscience Center of Excellence, Louisiana State University Health Sciences Center, New Orleans, LA 70112, USA; 6 Dental and Craniofacial Biology Center of Excellence, Louisiana State University Health Sciences Center, New Orleans, LA 70119, USA

**Keywords:** voltage-gated sodium channels, VGSCs, Na^+^,K^+^-ATPase, sodium pumps, cell cycle

## Abstract

Voltage-gated sodium channels (VGSCs) are the target for many therapies. Variation in membrane potential occurs throughout the cell cycle, yet little attention has been devoted to the role of VGSCs and Na^+^,K^+^-ATPases. We hypothesized that in addition to doubling DNA and cell membrane in anticipation of cell division, there should be a doubling of VGSCs and Na^+^,K^+^-ATPase compared to non-dividing cells. We tested this hypothesis in eight immortalized cell lines by correlating immunocytofluorescent labeling of VGSCs or Na^+^,K^+^-ATPase with propidium iodide or DAPI fluorescence using flow cytometry and imaging. Cell surface expression of VGSCs during phases S through M was double that seen during phases G0–G1. By contrast, Na^+^,K^+^-ATPase expression increased only 1.5-fold. The increases were independent of baseline expression of channels or pumps. The variation in VGSC and Na^+^,K^+^-ATPase expression has implications for both our understanding of sodium’s role in controlling the cell cycle and variability of treatments targeted at these components of the Na^+^ handling system.

## 1. Introduction

The modulation of voltage-gated sodium channels (VGSCs) and/or Na^+^,K^+^-ATPases (sodium pumps) plays a key role in the expression and treatment of several disease states including neuropathic pain [1], cancer [2,3,4], hypertension [5], and epilepsy [6]. For example, mutations in the SCN-9A gene that encodes the Na_V_1.7 VGSC are responsible for erythromelalgia, [7] paroxysmal extreme pain disorder, and congenital insensitivity to pain [8,9,10]. Over-expression of VGSCs and Na^+^,K^+^-ATPases in aggressive carcinomas enhances their ability to invade normal tissues and metastasize, but also makes them suitable targets for cancer therapy [11,12,13]. In addition, dysregulation of Na^+^,K^+^-ATPases contributes to disease states such as epilepsy, hypoglycemia or ischemia [14]. Several technologies designed to selectively target the over- or under-expression of sodium channels and sodium pumps to better manage these debilitating diseases have been proposed. Because VGSC and Na^+^,K^+^-ATPase expression varies based on the status of the organism, cellular environment, and on the stage of disease evolution, an understanding of the dynamics of underlying function and change in expression is essential for developing effective therapeutics and determining the optimum timing for their delivery.

We have known for decades that ion channels and shifts in membrane potential play important roles in the cell cycle that are responsible for preparing the cell for the sequence of events that must take place to ensure maintenance, replication and survival, through control of constantly changing intra- and extracellular ion concentrations [15]. For example, during the G1/S transition, the membrane potential is hyperpolarized due to influx of sodium and efflux of potassium [15]. Depolarization occurs during the G2/M transition caused by efflux of chloride ions [16]. During the G2/M transition, there is an increase in activity of potassium channels, which conduct K+ out of the cell, leading to a decrease in intracellular potassium, resulting in hyperpolarization [15,17]. In addition, the increase of intracellular calcium has been noted for its involvement with nuclear envelope breakdown, metaphase-anaphase transition, and cytokinesis [17]. Interestingly, signaling events associated with the role of potassium, calcium and chloride in the cell cycle have been studied extensively, but in light of the importance of VGSCs and Na^+^,K^+^-ATPases in membrane excitation and the maintenance of the electrochemical gradient in excitable cells, it is surprising that less is known about the role of VGSCs and Na^+^,K^+^-ATPases in modulating membrane potential and coordinating function during the cell cycle.

What is known is that shifts in the cell membrane potential are essential for cell cycle progression and that VGSCs and Na^+^,K^+^-ATPase play an important role in this process [15]. The opening of VGSCs that allows for the entry of sodium into the cell and depolarization of the cell membrane is required for transition from G0 to G1 [15]. Sodium pumps return sodium ions to the extracellular space to restore the resting membrane potential and the intracellular sodium concentration. Re-establishing the electrochemical gradient across the cell membrane is necessary for the cell to progress through S phase [15]. Importantly, in cancer cells that overexpress VGSCs, the resting potential of the membrane correlates with the expression level of cell surface VGSCs [18].

In spinal cord astrocytes, the influx of sodium achieves a more than four-fold increase of intracellular sodium in S phase-arrested cells compared to non-arrested controls [18]. It is not clear whether the excess Na^+^ concentration is due to an upregulation in the number of cell surface VGSCs, an increase in the frequency of channel opening or an inhibition of sodium pump function [19,20,21]. Treating spinal astrocytes with tetrodotoxin, a VGSC blocker, has no effect on cell proliferation, but treating with low doses of ouabain, a Na^+^,K^+^-ATPase inhibitor, increases the proportion of quiescent cells in S phase [18]. This information taken together is evidence that an increase or decrease of extracellular potassium, an increase of intracellular sodium, or membrane depolarization are important contributors to astrocyte proliferation.

Electrophysiological recording revealed that in many forms of cancer, depolarization initiates robust sodium currents [21]. Additionally, Coombs et al. discovered that there is an initial increase in sodium currents immediately after depolarization [20]. Sodium currents remain elevated through meiosis and the first cleavage. The increase in sodium currents is evidence for an increase in VGSC activity in S through M phases.

Aggressive carcinomas over-express VGSCs compared to their non-cancerous or stromal counterparts [4,22], but even in highly malignant cancers, there is relative heterogeneity in VGSC expression in cells within a given solid tumor that fall into 2 groups. The cells with the highest level of expression comprise 30–45% of the total. We hypothesized that the most highly expressing cells are those in the S-M phase of the cell cycle, whereas the cells that express relatively fewer VGSCs are in the G0-G1 phase. Accordingly, we assessed the relative levels of VGSC and Na^+^,K^+^-ATPase protein expression in eight immortalized cancer and noncancerous cell lines at phases of the cell cycle in which there are single (G0–G1) or double (S-M) copies of DNA using a flow cytometry paradigm and confirmed with confocal imaging. We used both noncancerous (low expression) and cancerous (high expression) cell lines to determine whether variation of cell-surface VGSC and Na^+^,K^+^-ATPase expression during the cell cycle is dependent upon the baseline expression of cells.

## 2. Materials and Methods

### 2.1. Cell Culture

We compared VGSC and Na^+^,K^+^-ATPase expression among different forms of cancer using human A549 lung and MDA-MB-231 breast cancer and H28 mesothelioma cells compared with the VGSC and Na^+^,K^+^-ATPase expression in their non-cancerous counterparts, MRC5 non-cancerous lung cells, MCF-10a non-cancerous breast cells and MeT-5a pleural effusion cells, respectively. Across-species comparisons for VGSC and Na^+^,K^+^-ATPase expression in malignant cells were also made using murine AB1 mesothelioma and 4T1 triple-negative breast cancer cells. Cell lines were selected so that cancer cell lines that overexpress VGSCs could be compared to a corresponding noncancerous cell line. While these non-cancerous cell lines have been immortalized, they do not have tumorigenic capacity. Moreover, noncancerous cells have been shown to have a resting membrane potential identical to primary cells from the same organ. All cell lines were purchased from American Type Culture Collection (www.atcc.org (accessed on 22 April 2022)), except AB1 cells, which were purchased from Millipore Sigma (www.sigmaaldrich.com (accessed on 22 April 2022)). A549 cells were cultured with F-12K supplemented with 10% Fetal Bovine Serum (FBS) (www.thermofisher.com (accessed on 22 April 2022)) and 1% penicillin/streptomycin (pen/strep; www.thermofisher.com (accessed on 22 April 2022)). MRC5 cells were cultured with Dulbecco’s Modified Eagle’s Medium (DMEM; www.thermofisher.com (accessed on 22 April 2022)) supplemented with 10% FBS and 1% pen/strep. H28, MDA-MB-231, 4T1 and AB1 cells were cultured with RPMI medium (www.atcc.org (accessed on 22 April 2022)) with 10% FBS, 1% pen/strep and 10 mM N-2-hydroxyethylpiperazine-N′-2-ethanesulfonic acid (HEPES; www.thermofisher.com (accessed on 22 April 2022)). MCF-10a cells were cultured with mammary epithelial growth medium (MEGM) media supplemented with 10% FBS, 1% pen/strep and MEGM kit (https://bioscience.lonza.com (accessed on 22 April 2022)). MeT-5a cells were cultured with M199 media (www.sigmaaldrich.com (accessed on 22 April 2022)) supplemented with 10% FBS, 1% pen/strep, 3.3 nM epidermal growth factor (EGF; www.sigmaaldrich.com (accessed on 22 April 2022)), 400 nM hydrocortisone (www.sigmaaldrich.com (accessed on 22 April 2022)), 870 nM bovine insulin (www.sigmaaldrich.com (accessed on 22 April 2022)), 20 mM HEPES and 0.1 mL trace elements (ecatalog.corning.com (accessed on 22 April 2022)). 

### 2.2. Antibody Conjugation

The pan-specific antibodies for VGSCs (www.alomone.com (accessed on 22 April 2022)) and Na^+^,K^+^-ATPases (www.abcam.com (accessed on 22 April 2022)) were conjugated to allophycocyanin (APC; Ex/Em: 594/633; biotium.com (accessed on 22 April 2022)) or to R-phycoerythrin (RPE; Ex/Em: 488–561/578; biotium.com (accessed on 22 April 2022)) using the Biotium Mix-N-Stain protocol. 

### 2.3. Flow Cytometry

**Figure 1:**
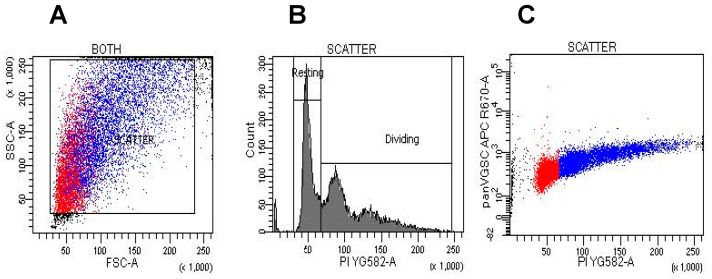
Gating single vs. double DNA label. This representative graph illustrates the gating for amount of DNA using PI on A549 cells in combination with a pan-VGSC antibody. All experiments were gated in the same way. Cells were first gated by forward scatter and side scatter, shown in (**A**). The resulting population was separated by the concentration of DNA label (PI or DAPI), indicating resting or dividing cells, Shown in (**B**). Due to the ubiquitous nature of VGSCs and Na^+^,K^+^-ATPases, all cells were positive for their antibody staining. (**C**) shows cells positive for pan-VGSC antibody divided into resting (blue) and dividing (red) groups.

Cells were cultured in T75 flasks to 80–90% confluence and harvested using 2–3 mL Corning CellStripper (ecatalog.corning.com (accessed on 22 April 2022)), fixed with 1% paraformaldehyde (PFA) for 10 min, blocked with 5% goat serum and 3% bovine serum albumin (BSA) solution for 1 h, and washed with phosphate-buffered saline (PBS). Becaues of the low PFA concentration, there are fewer covalent bond crosslinks than the standard 4% concentration. This fixing protocol allows all antibodies to permeate the cell membrane without detergent permeablization. Cells were then incubated in APC- or PE-conjugated antibodies at 1:50 in PBS overnight at 4 °C protected from light. The cells were then washed once in PBS and resuspended in 0.5 µg/mL propidium iodide (PI; www.bdbiosciences.com (accessed on 22 April 2022)) or 1 µg/mL 4′,6-diamidino-2-phenylindole (DAPI; www.thermofisher.com (accessed on 22 April 2022)). The choice of nuclear stain was based on the emission of the conjugated antibody. DNA content vs. VGSC or Na^+^,K^+^-ATPase label expression were measured on BD Fortessa or BD FACSCanto II flow cytometers at low flow rates (instrument configurations are listed in Supplemental Tables S1 and S2). Approximately 25,000 cells were acquired for each sample. Doublets and debris were gated out and DNA content (DAPI or PI) was plotted against APC or RPE fluorescence (full gating strategies are shown in Figure 1). Gates were defined for cells in G0-G1 phases (low DNA fluorescence) and S-M phases (high DNA fluorescence) groups, and the median fluorescent intensity (MFI) of their respective APC or RPE fluorescence was measured. There were three replicates of each experiment. Pre-blocked controls resulted in only 0.65% of cells labeling positive.

### 2.4. Statistical Analysis

For each cell line, the median APC fluorescence (VGSCs) was determined for G0-G1 and S-M phases. To determine relative cell surface expression in these phases, the mean of the three determinations of median fluorescence intensity (MFI) for S-M cells was divided by the mean of the three determinations of MFI for the G0–G1 phases. The relative cell surface expression of Na^+^,K^+^-ATPase was determined for each cell line in the same manner, using the mean of three MFI and difference among the MFIs analyzed using one-way ANOVAs.

### 2.5. Immunocytofluorescence

As a verification of our flow cytometry data, we imaged cells for VGSCs and Na^+^,K^+^-ATPase counterstained with an anti-phospho-histone H3 Ser10 (pH3ser10) antibody to identify cells in the M phase. Cells were incubated in the mitotic agent S-trityl-L-cysteine (STLC; 50 µM) for 24 h prior to harvest to increase the incidence of mitotic cells. Cells were then harvested using CellStripper and fixed with 1% PFA for 10 min, then blocked and permeabilized with 3% BSA 5% goat serum 0.25% TritonX (www.thermofisher.com (accessed on 22 April 2022)) for 1 h. Groups of cells were labeled with the pan-specific anti-VGSCs antibody and the pan-specific anti-Na^+^,K^+^-ATPase antibody either through sequential individual staining or using conjugated antibodies. Conjugated antibodies were used at a concentration of 1:50 and the rabbit anti- pH3ser10 antibody (www.cellsignal.com (accessed on 22 April 2022)) was used at a concentration 1:100 and incubated for 2 h at room temperature, shielded from light. Cells were washed with PBS and then incubated with an anti-rabbit secondary antibody Alexa Fluor 488 (www.abcam.com (accessed on 22 April 2022)) or Alexa Fluor 555 (www.abcam.com (accessed on 22 April 2022)), both at a concentration of 1:1200. Cells were incubated at room temperature for 1 h protected from light then washed with PBS. To label for nuclei, cells were incubated in 1:1000 NucSpot488 (biotium.com (accessed on 22 April 2022)) for 10 min or 1:600 Draq5 (www.thermofisher.com (accessed on 22 April 2022)) for 6 min then washed with PBS. Coverslips were affixed with ProLong Gold. Slides were imaged on a Leica SP8 Confocal Microscope using lasers 488, 514 or 561, and 633 nm.

## 3. Results

**Figure 2:**
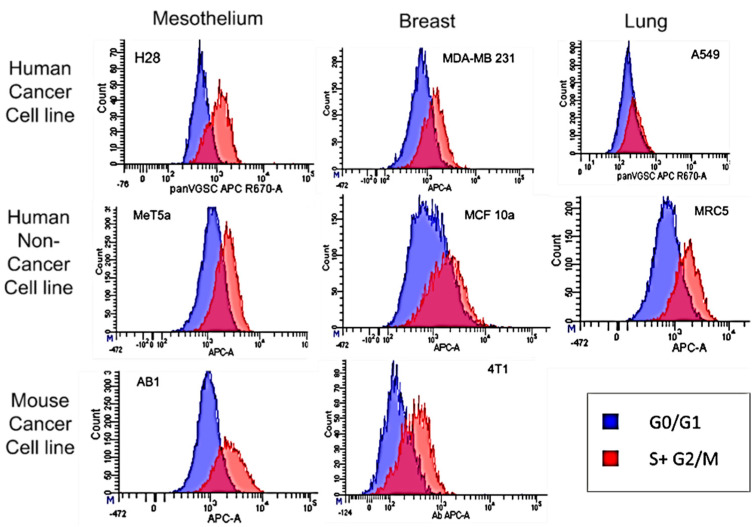
VGSC protein expression of cells in G0/G1 vs. S-M phases. Cells labeled with PI and a pan-specific VGSC antibody conjugated to APC were analyzed using flow cytometry. The number of APC fluorescence events per cell were compared in cells with low or high PI fluorescence. Cells gated for 2 copies of DNA (high PI fluorescence; red) have significantly greater median fluorescent intensity than cells gated for 1 copy of DNA (low PI fluorescence; blue). Graphs shown are representative of triplicate experiments.

A representative graph of VGSC cell surface expression in G0-G1phases vs. S-M phases from each cell line is depicted in Figure 2. Scatter plots for no label, VGSC label only, DNA label only, DNA label + VGSC label, gating strategy and label controls are depicted in Supplementary Figures S1–S8. Interestingly, Non-random peaks of DNA labelling appears to show that some dividing MCF-10a cells may be producing double, triple and even quadruple DNA (Figure S5). This questions the normality of this cell line that is frequently used as a “normal” breast control.

Representative graphs of Na^+^,K^+^-ATPase cell surface expression in G0-G1phases vs. S-M phases from each cell line are depicted in Figure 3. Scatter plots for no label, VGSC label only, DNA label only, DNA label + VGSC label, gating strategy and label controls are depicted in Supplementary Figures S9–S16. 

**Figure 4:**
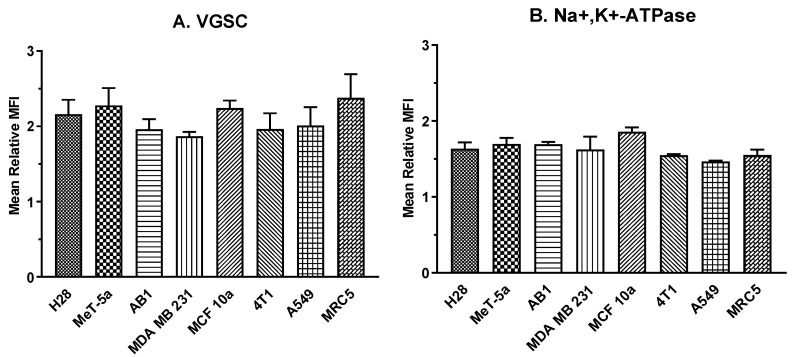
Mean shift in median VGSC (**A**) and Na^+^,K^+^-ATPase (**B**) expression in the eight cell lines. Bars represent the mean fold difference between median fluorescent intensities of cells with 2 copies of DNA and median fluorescent intensities of cells with one copy of DNA. One-way ANOVAs revealed that there was no significant difference among the cell lines for either VGSCs or Na^+^,K^+^-ATPase. Error bars are representative of the standard deviation of separate flow cytometry experiments; N = 3.

For each replicate, MFI gated for VGSCs in cells with single copy DNA (G0–G1 phases) and double copy DNA (S-M phases) was determined. For each cell line, the MFI in S-M phase cells was significantly greater than VGSC protein expression in G0–G1 phase cells (*p* < 0.01), with the mean MFI for cells in S-M phases was between 1.86- and 2.38-fold greater than that of cells in G0-G1 phases ($\overset{¯}{X}$ = 2.11 ± 0.065) across all 8 cell lines (Figure 4A). A one-way ANOVA showed no main effect of relative expression across the eight cell lines.

For all cell lines, MFI for Na^+^,K^+^-ATPase protein expression in S-M phase cells was significantly greater than for Na^+^,K^+^-ATPase protein expression in G0–G1 phase cells with the MFI for Na^+^,K^+^-ATPase in cells in S-M phases was between 1.47 and 1.86 times greater than for that of cells in G0-G1 phases (Figure 4B), with a mean across all 8 cell lines of $\overset{¯}{X}$ = 1.64 ± 0.04. The relationship between VGSC and Na^+^,K^+^-ATPase expression and the phase groupings of the cell cycle were not significantly different whether the cell line was from a human or murine source or were expressed in cancerous or non-cancerous cells. 

**Figure 5:**
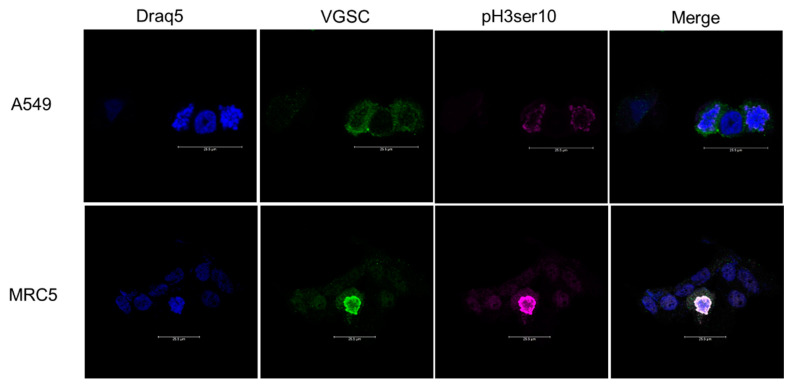
Immunocytofluorescence of cancerous (A549) and noncancerous (MRC5) lung cells stained for pan-VGSCs and pH3ser10. Six fields were analyzed for each cell line and condition. The first column shows cells stained with Draq5 alone. Column 2 shows cells labeled with pan-VGSC antibody. Column 3 shows cells labeled with anti-pH3ser10. The final column shows all channels merged together. The top row of images is representative of cell populations for A549 cells and the bottom row of images is representative of the cell population for MRC5 cells. Of the four A549 cells, two are positive for the mitotic marker pH3ser10. These two cells positive for pH3ser10 also have a greater VGSC fluorescence. The MRC5 cell population imaged above contains eight individual cells. However, only one is positive for pH3ser10. The cell positive for pH3ser10 also has greater fluorescence associated with VGSC expression. Scale bar represents 25.5 microns.

In addition to flow cytometry data providing evidence that dividing cells have double the number of sodium channels and 1.5 times the number of Na^+^,K^+^-ATPases than resting cells, immunocytofluorescence images confirm this finding. Cells positive for pH3ser10 exhibited greater VGSC fluorescence than pH3ser10 negative cells (Figure 5). 

**Figure 6:**
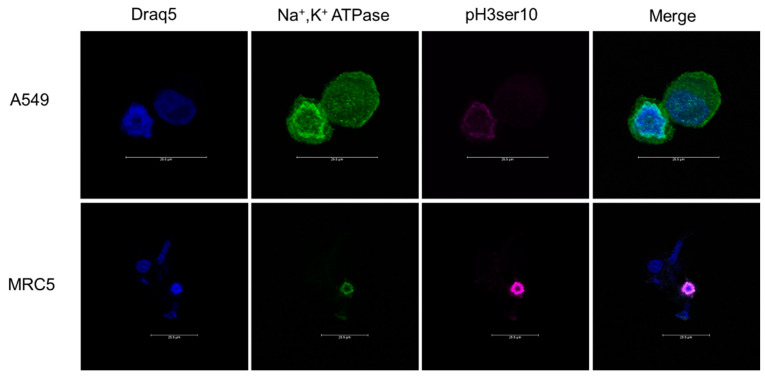
Immunocytofluorescence of cancerous (A549) and noncancerous (MRC5) lung cells stained for pan-Na^+^,K^+^-ATPase and pH3ser10. Six fields were analyzed for each cell line and condition. The first column shows cells stained with Draq5 alone. Column 2 shows cells labeled with pan-Na^+^,K^+^-ATPase antibody. Column 3 shows cells labeled with anti-pH3ser10. The final column shows all channels merged together. The top row of images is representative of cell populations for A549 cells and the bottom row of images is representative of the cell population for MRC5 cells. Of the two cells imaged in the A549 cell population, only one cell is positive for the pro-mitotic marker pH3ser10. This cell positive for pH3ser10 also shows greater fluorescence associated with Na^+^,K^+^-ATPase expression. Similarly, there is only one cell positive for pH3ser10 in the MRC5 population photographed. The cell expressing pH3ser10 also shows an abundance of fluorescence associated with Na^+^,K^+^-ATPase expression. Scale bar represents 25.5 microns.

Similarly, Na^+^,K^+^-ATPase images of pH3ser10-positive cells exhibited greater Na^+^,K^+^-ATPase fluorescence than pHser10-negative cells (Figure 6). 

## 4. Discussion

With this series of experiments, we demonstrated that the expression of cell-surface VGSCs doubles during cell division compared to G0/G1 phases of the cell cycle. Similarly, cell-surface expression of Na^+^,K^+^-ATPase increases by 1.5-fold during the stages of cell division when there is a doubling of DNA. These increases occurred irrespective of the baseline expression of these sodium-handling proteins. In addition, the upregulation occurred in cancerous and noncancerous cells lines derived from three different source organs.

It is tempting to simplify these results as VGSC expression doubling because DNA doubles. However, there are far too many modulators of both RNA and protein expression for a direct one-to-one interpretation. Moreover, the DNA for Na^+^,K^+^-ATPase also doubles, yet the protein increases only by 1.5-fold. Many proteins critical to the cell cycle up- or down-regulate during specific cycle phases. Thus, we propose that our findings of a 2-fold increase for VGSCs and a 1.5-fold increase for Na^+^,K^+^-ATPase may be important for our understanding of the progression through cell division.

In addition, other researchers have shown variation of activity and expression of potassium and calcium channels throughout the cell cycle, but changes in sodium handling has only been implicated by changes in membrane potential [15]. VGSC and Na^+^/K^+^ ATPase cell surface protein expression had not been investigated. Thus, the question of whether the modulation of membrane potential due to either a change in firing frequency, a downregulation of Na^+^,K^+^-ATPase, or an upregulation of VGSC remained. While our results do not directly demonstrate that these channels and pumps are functional, there is considerable literature to show that they are [3,5,7,11,19,20,21,22]. Thus our results are consistent with the interpretation that the relative increased expression of VGSCs vs. Na^+^,K^+^-ATPases at various phases of the cycle are certainly involved. 

Furthermore, these results are likely to be important for our understanding of the mechanism or variability in efficacy of current and proposed treatments of diseases that involve modulation of sodium channels and/or sodium pumps. Modulation of VGSC subtype distribution and expression has been reported in many genetic and iatrogenic disease states. For example, changes in VGSC expression and distribution have been correlated with alterations in pain perception associated with peripheral nerve injury and inflammation [1] and more recently with inherited channelopathies, such as inherited primary familial erythromelalgia, paroxysmal extreme pain disorder and congenital insensitivity to pain [8,9,10,23,24,25]. VGSC dysregulation has also been implicated in cases of essential hypertension [5] and epilepsy [6]. In addition, alterations in Na^+^,K^+^ -ATPase expression and function has been associated with hypertension, inflammation [25,26] and the control of insulin and the development of painful diabetic polyneuropathy [1,27]. Most recently, VGSCs expression has been shown to be directly correlated with tissue invasion, malignancy and metastasis in many forms of cancer [2,3,4,11,12,13,18,20,28,29,30] and Na^+^,K^+^-ATPase has been shown to play a role in oncogenesis, tumor growth and development and metastasis [31]. It has been postulated that fluctuation in the membrane potential produced by channel opening and pump activity is integral in these functions that give neoplastic cells an evolutionary advantage [29,32].

The observed changes in VGSC and Na^+^,K^+^-ATPase expression seen in the present study may provide the basis for a more thorough understanding of the sodium handling system in cell replication and disease, a focus for seeking targeted treatments and a guide for optimizing the timing of therapeutic intervention.

## Figures and Tables

**Figure 3:**
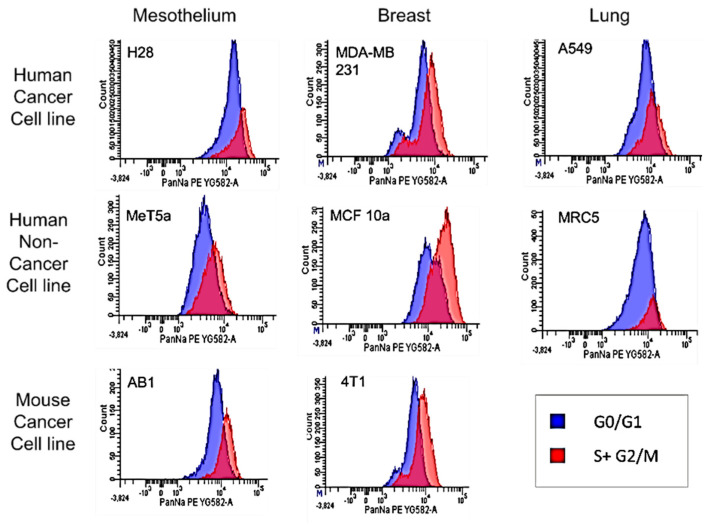
Na^+^,K^+^-ATPase protein expression of cells in G0/G1 vs. S-M phases: Cells that were labeled with pan-specific Na^+^,K^+^-ATPase antibody conjugated to RPE and DAPI were analyzed using flow cytometry. The number of RPE fluorescence events per cell were compared in cells with low or high DAPI fluorescence. Cells gated for two copies of DNA (high DAPI fluorescence; red) have significantly greater median fluorescent intensity than cells gated for 1 copy of DNA (low DAPI fluorescence; blue). Graphs shown are representative graphs of triplicate experiment.

## Data Availability

All reasonable requests for the flow cytometry data files will be honored. Send requests to Dennis Paul, Ph.D. Dept. of Pharmacology, LSU Health Sciences Center New Orleans, 1901 Perdido St. New Orleans, LA 70112, U.S.A.

## Supplementary Materials

The following supporting information can be downloaded at: https://www.mdpi.com/article/10.3390/cells11203240/s1, Table S1: BD Fortessa Configuration; Table S2: BD Canto Configuration.; Figures S1–S8: VGSC-APC + PI Flow Cytometry Plots; Figures S9–S16: Na^+^,K^+^-ATPase-RPE + DAPI Flow Cytometry Plots.
