# Supplementary material for: Effect of Cell Cycle on Cell Surface Expression of Voltage-Gated Sodium Channels and Na+,K+-ATPase
Source: Cells. 2022 Oct 15;11(20):3240. doi: 10.3390/cells11203240 (PMC9600173; doi:10.3390/cells11203240)
Supplement: Supplementary file 1 [file cells-11-03240-s001.zip › cells-1817813-supplementary.pdf]

Supplemental Materials

Table S1. BD Fortessa Configuration

Cytometer Configuration												
Software Version	BD FACSDiva 9.0											
Export Time	12/15/2020 11:39											
User												
Cytometer	LSRFortessa											
Serial Number	1											
Configuration Name	Universal MiFloCyte											
Folder Name	Current Configuration											
Comments												
Window Extension	10											
Laser Name	Type	Waveleng	Power	Detector A	Detector	Channel	Mirror	Filter	Paramete	Fsc Chann	Reference	Position
Blue	Blue	488	50	Octagon	A	3	685 LP	710/50 BP	B710	0	Y	1
					B	2	505 LP	530/30 BP	B530			
					C	1		488/10 BP	SSC			
					D							
					E							
					F							
					G							
					H							
Red	Red	640	40	Octagon	A	9	750 LP	780/60 BP	R780			3
					B	8	690 LP	730/45 BP	R730			
					C	7		670/14 BP	R670			
					D							
					E							
					F							
					G							
					H							
Violet	Violet	405	50	Octagon	A	6	600 LP	610/20 BP	V610			2
					B	5	505 LP	525/50 BP	V525			
					C	4		450/50 BP	V450			
					D							
					E							
					F							
					G							
					H							
561 Yellow-Green	Custom	561	0	Octagon	A	14	750 LP	780/60 BP	YG780			4
					B	13	685 LP	710/50 BP	YG710			
					C	12	635 LP	670/30 BP	YG670			
					D	11	600 LP	610/20 BP	YG610			
					E	10		582/15 BP	YG582			
					F							
					G							
					H							

Table S2. BD Canto Configuration

Cytometer Configuration												
Software Version	BD FACSDiva 6.1.3											
Export Time	6/1/2011 11:06											
User												
Cytometer	FACSCantoII											
Serial Number	V96300106											
Configuration Name	3-laser, 8-color (4-2-2) HTS											
Folder Name	LSU Configuration											
Comments												
Window Extension	7											
Laser Name	Type	Wavelength	Power	Detector Array	Detector	Channel	Mirror	Filter	Parameter	Fsc Channel	Reference	Position
Blue	Blue	488	20	Octagon	A	1	735 LP	780/60 BP	PE-Cy7	0	Y	2
					B	2	655 LP	670 LP	PerCP-Cy5-5			
									PerCP			
					C		610 LP					
					D	3	556 LP	585/42 BP	PE			
					E	4	502 LP	530/30 BP	FITC			
					F	5		488/10 BP	SSC			
					G							
					H							
Red	Red	633	17	Trigon	A	6	735 LP	780/60 BP	APC-Cy7			3
					B		685 LP					
					C	7		660/20 BP	APC			
Violet	Violet	405	25	Trigon	A	9	502 LP	510/50 BP	AmCyan			1
					B	8		450/50 BP	Pacific Blue			
					C							

H28: VGSCs-APC + PI

Vehicle Only:

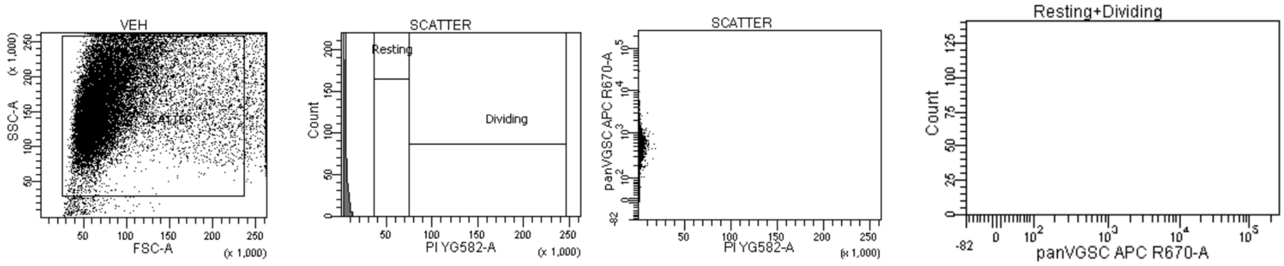

VGSC label only:

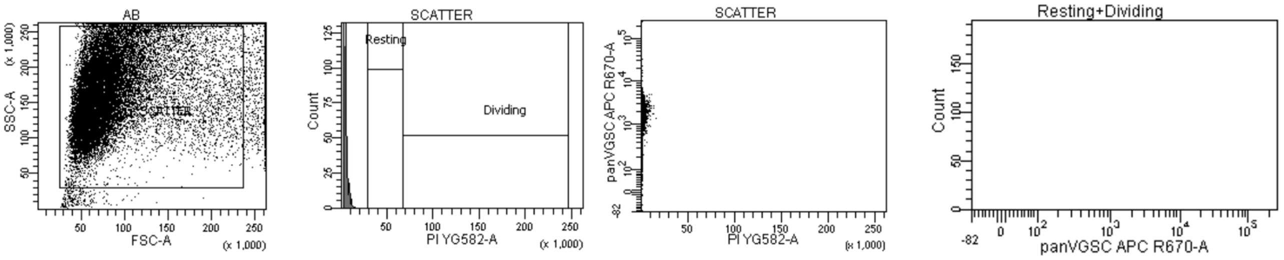

DNA label only:

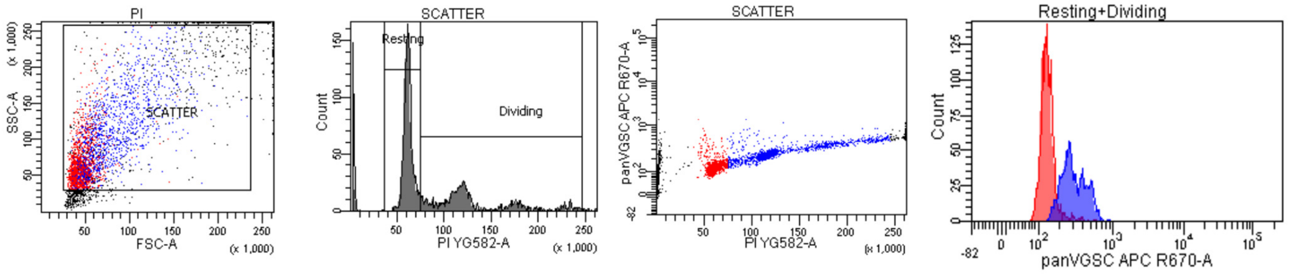

DNA label + VGSC label:

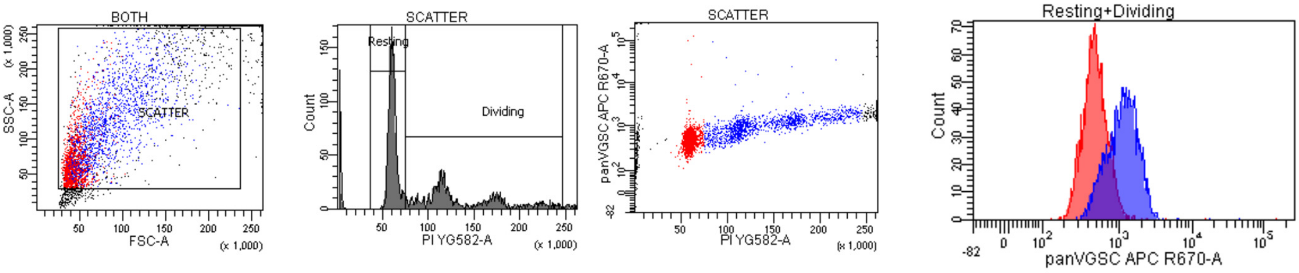

Figure S1. H28 VGSC-APC + PI Flow Cytometry Plots

MeT-5a: VGSCs-APC

Vehicle Only:

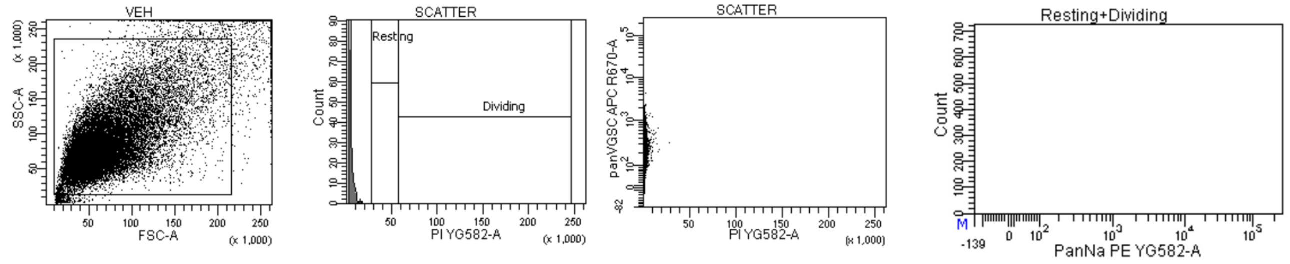

VGSC label only:

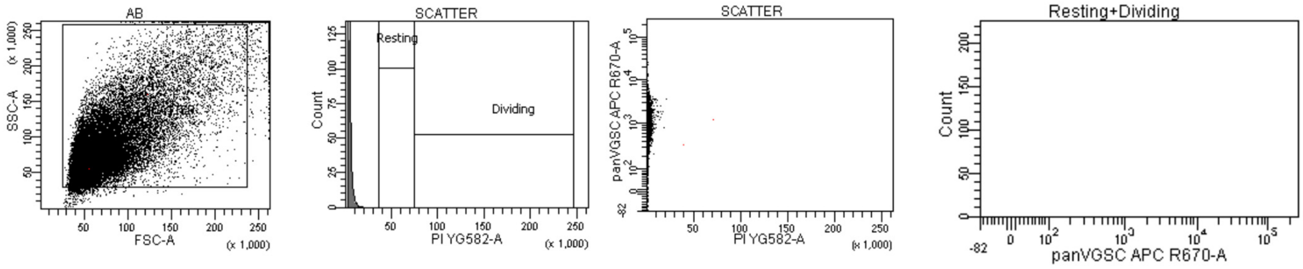

DNA label only:

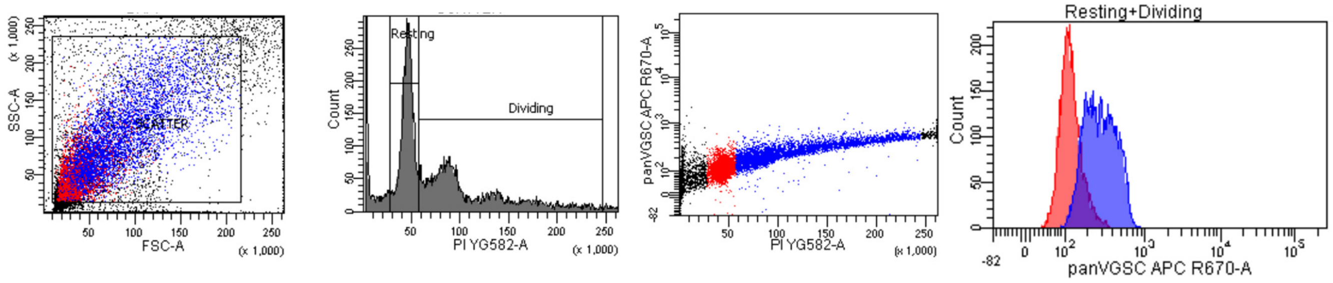

DNA label + VGSC label:

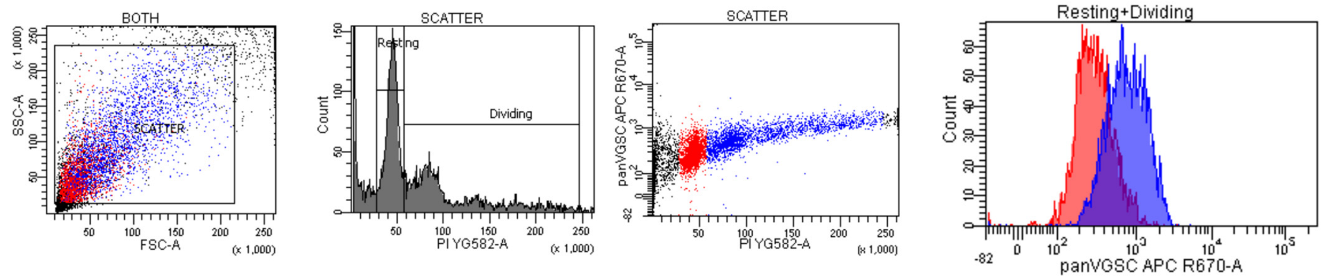

Figure S2. MeT-5a VGSC-APC + PI Flow Cytometry Plots

AB1: VGSCs-APC + PI

Vehicle Only:

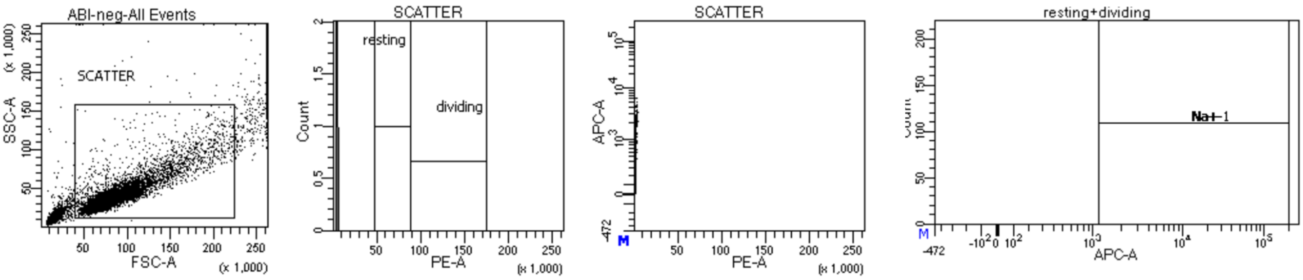

VGSC label

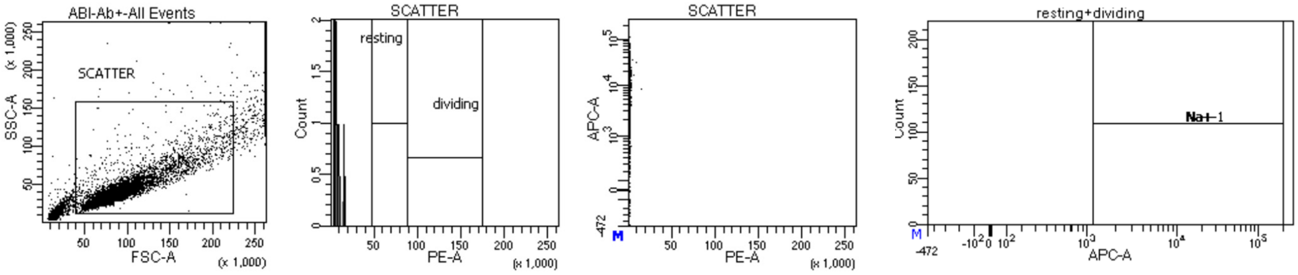

DNA label only:

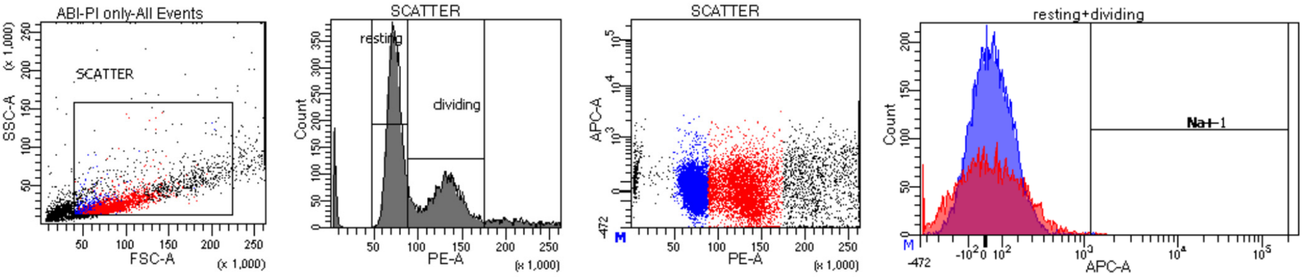

DNA label + VGSC label:

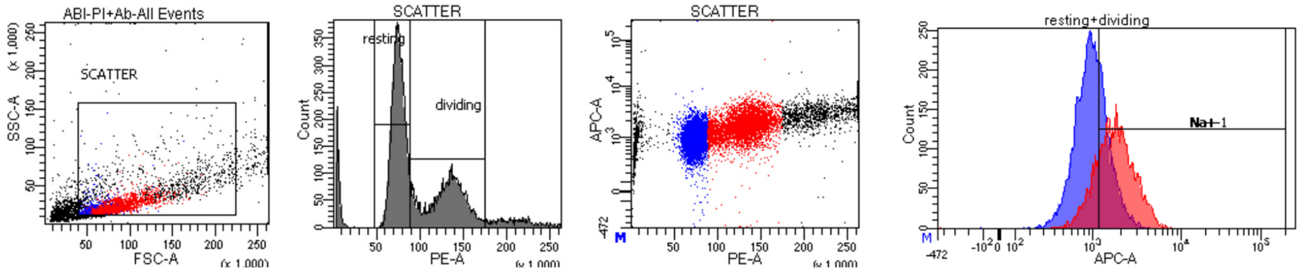

Figure S3. AB1 VGSC-APC + PI Flow Cytometry Plots

MDA-MB-231: VGSCs-APC + PI

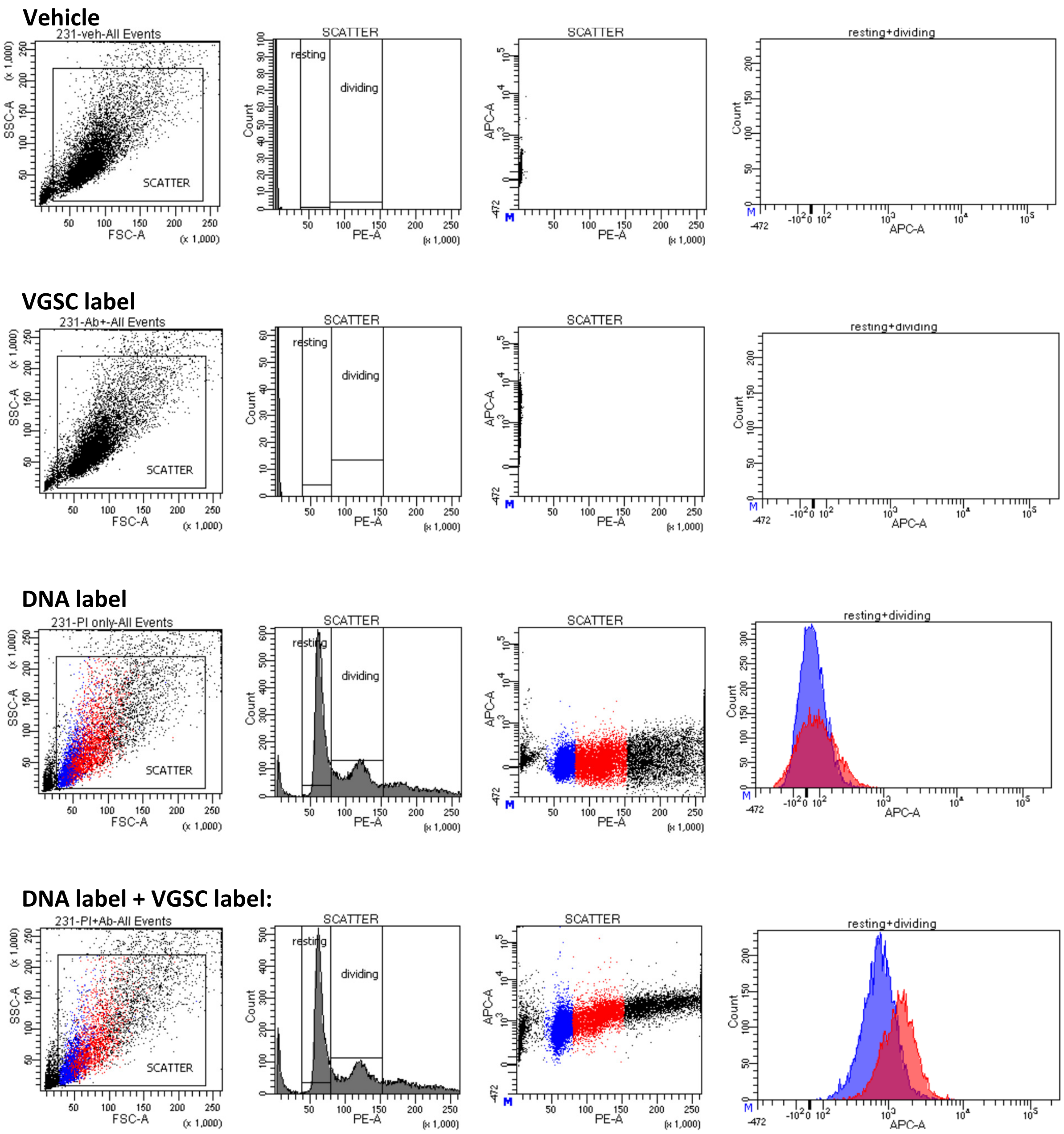

Figure S4. MDA-MB-231 VGSC-APC + PI Flow Cytometry Plots

MCF-10a: VGSCs-APC + PI

Vehicle Only:

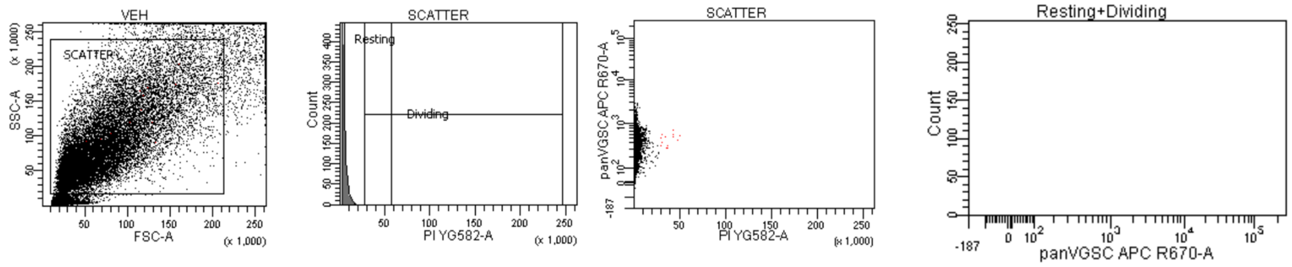

VGSC label

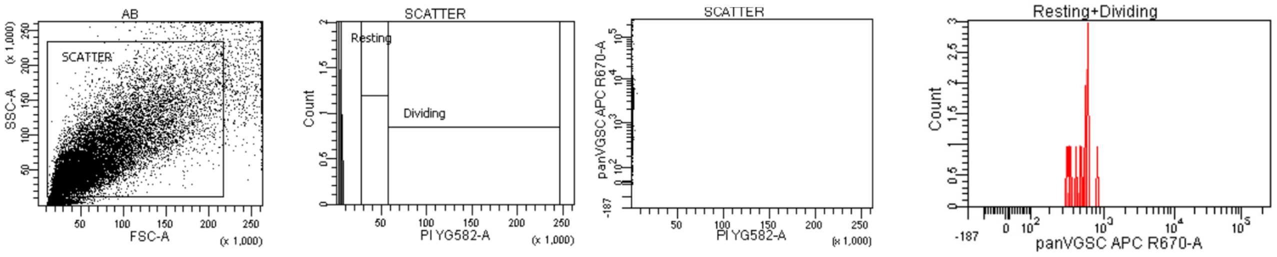

DNA label only:

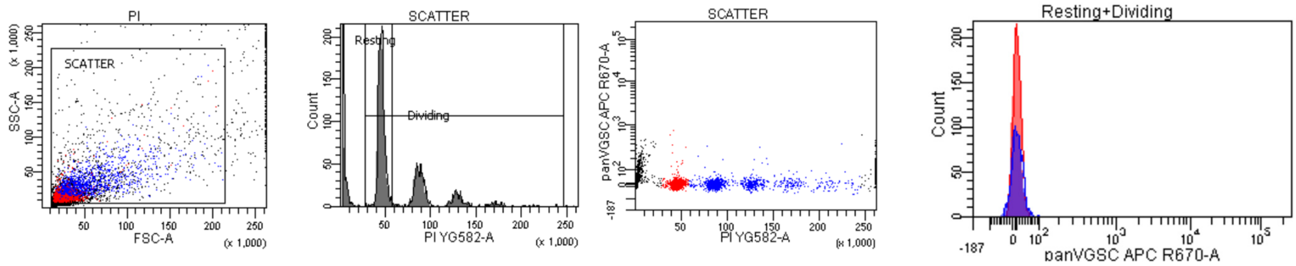

DNA label + VGSC label:

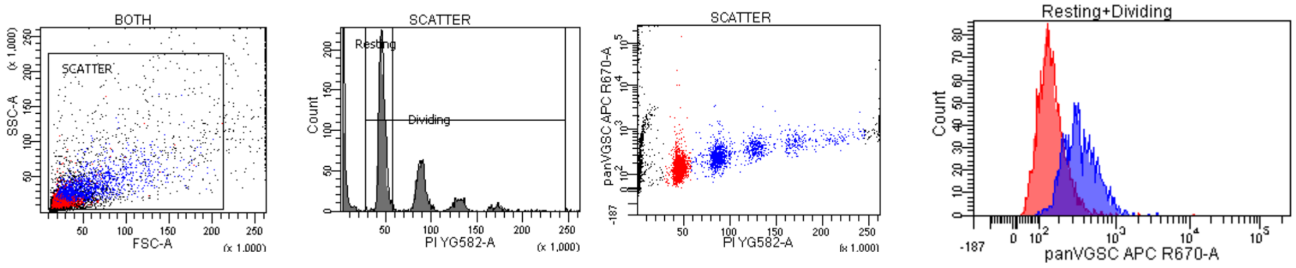

Figure S5. MCF-10a VGSC-APC + PI Flow Cytometry Plots

4T1: VGSCs-APC + PI

Vehicle Only:

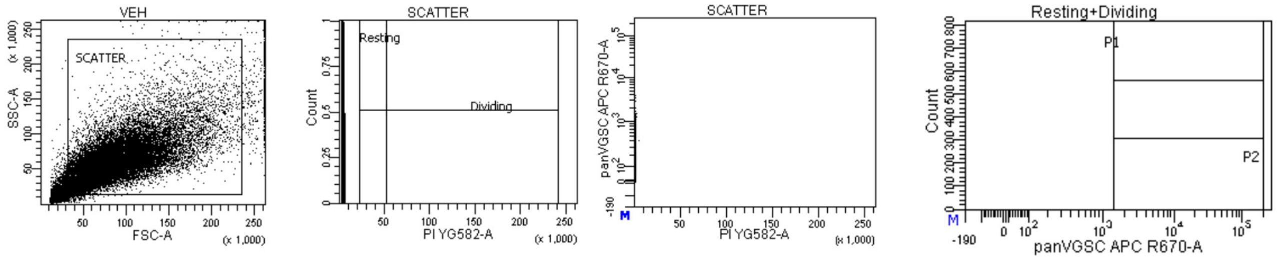

VGSC label

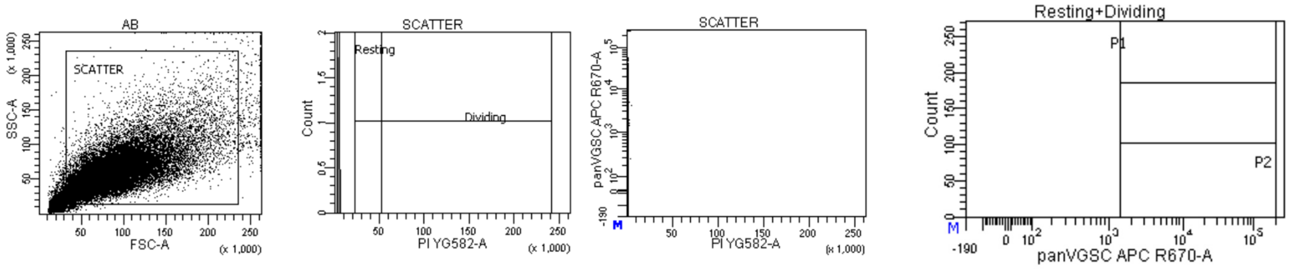

DNA label only:

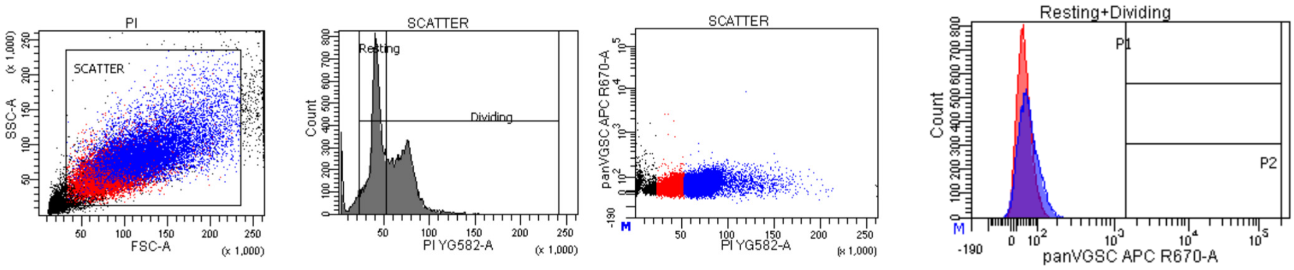

DNA label + VGSC label:

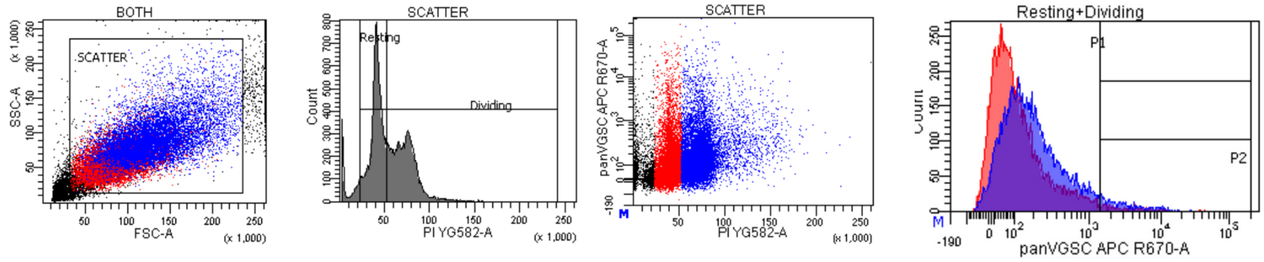

Figure S6. 4T1 VGSC-APC + PI Flow Cytometry Plots

A549: VGSCs-APC + PI

Vehicle Only:

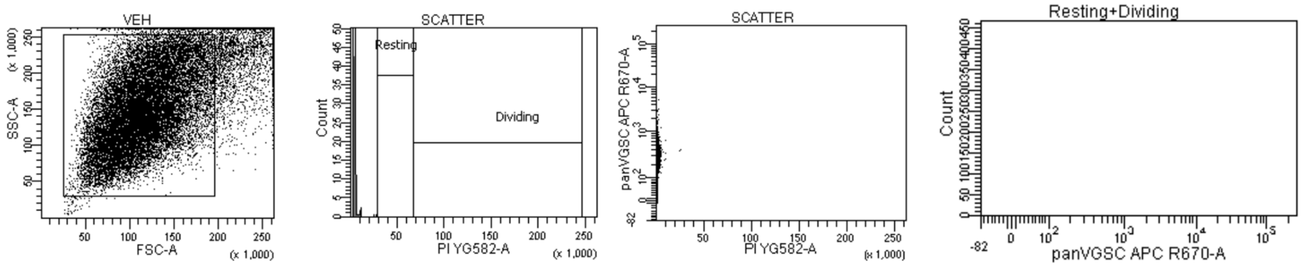

VGSC label only:

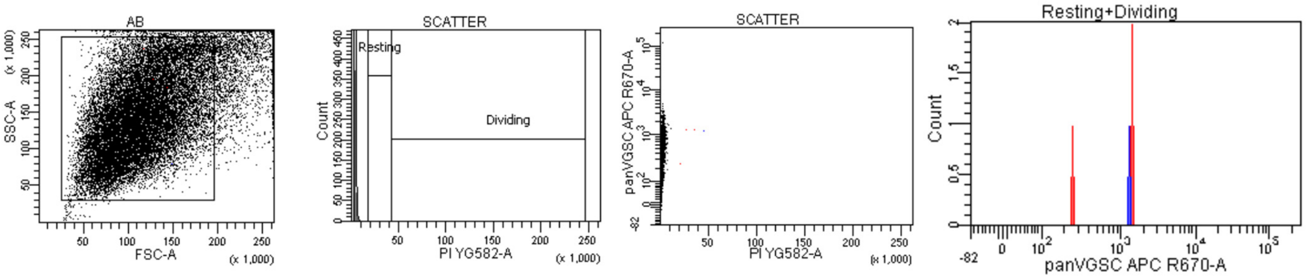

DNA label only:

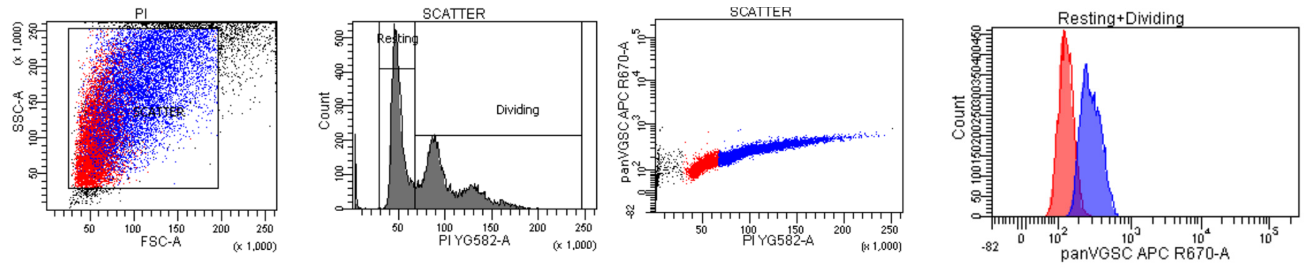

DNA label + VGSC label:

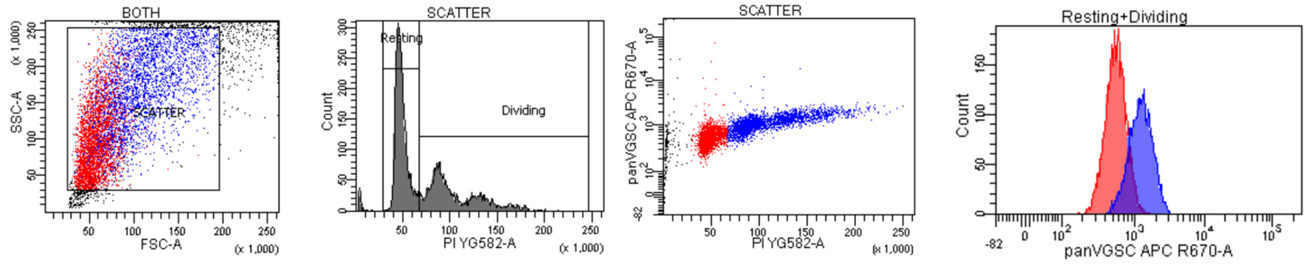

Figure S7. A549 VGSC-APC + PI Flow Cytometry Plots

MRC5: VGSCs-APC + PI

Vehicle Only:

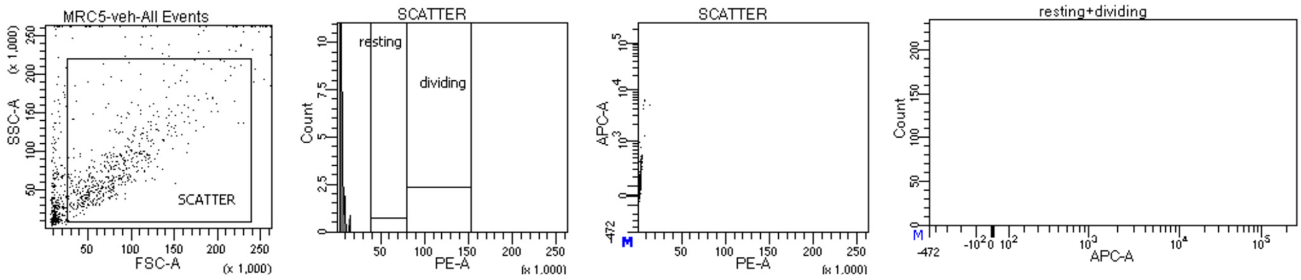

VGSC label

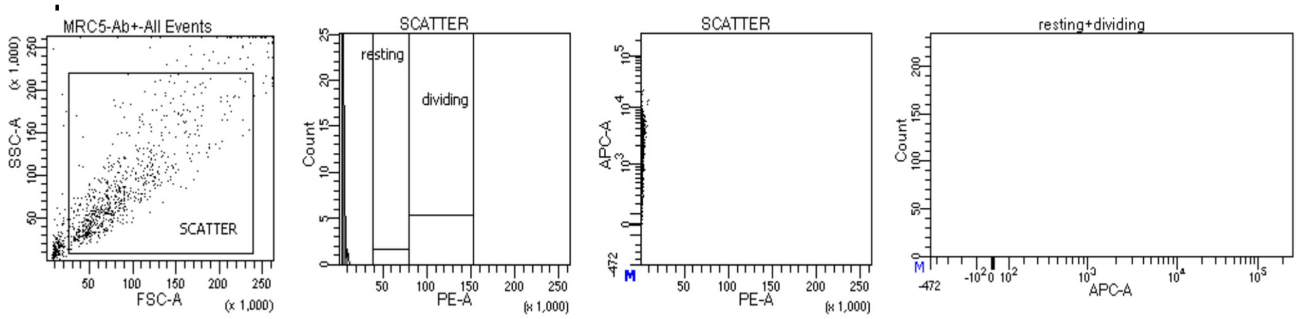

DNA label only:

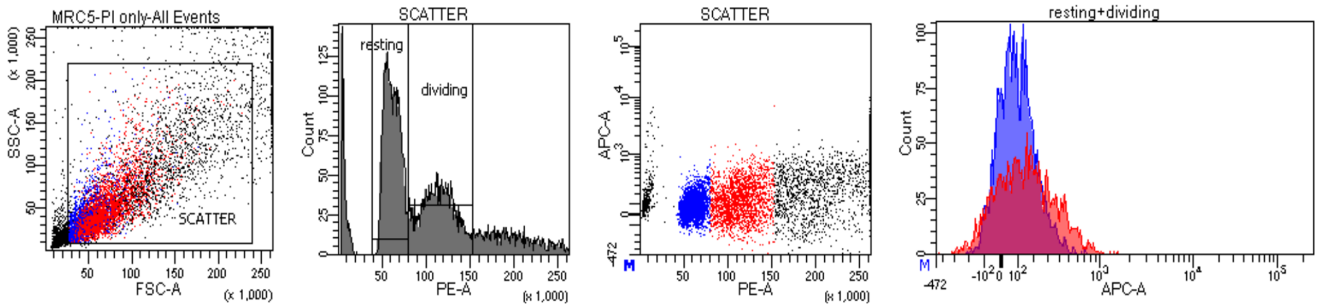

DNA label + VGSC label:

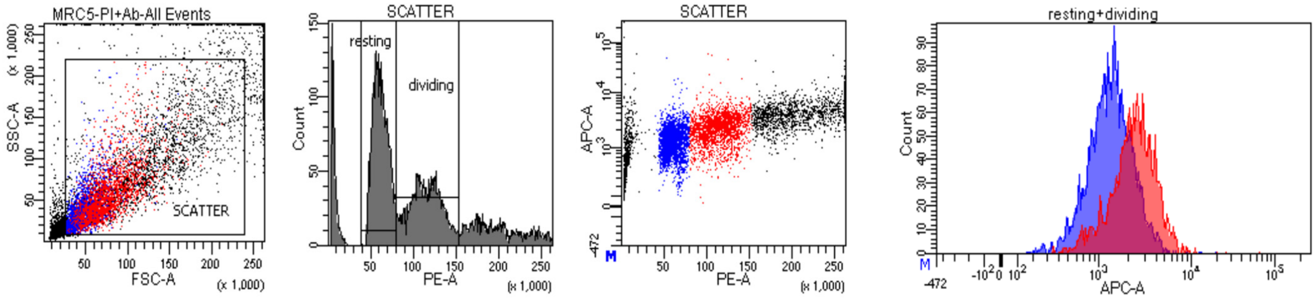

Figure S8. MRC5 VGSC-APC + PI Flow Cytometry Plots

H28: Na,K-ATPase -PE + DAPI

Vehicle Only:

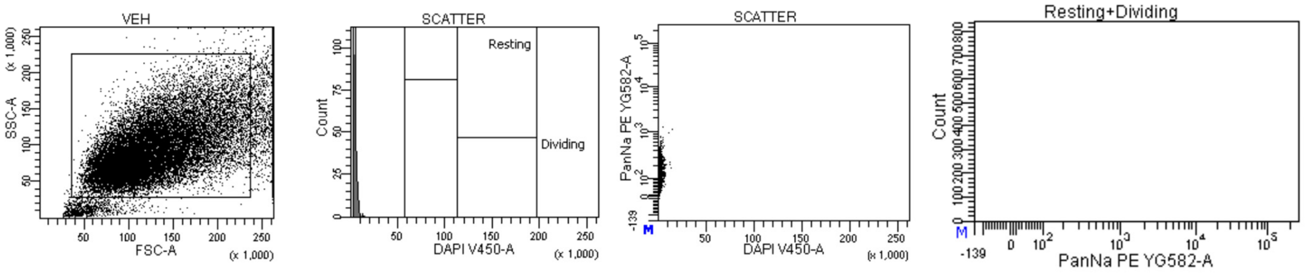

Na,K-ATPase labeled only:

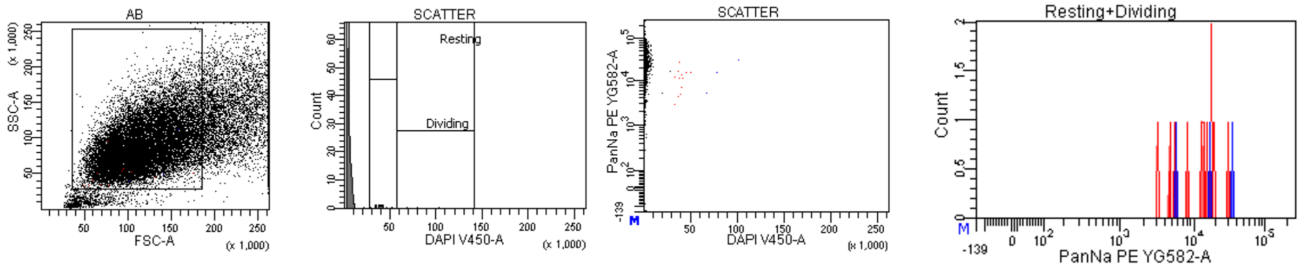

DNA label only:

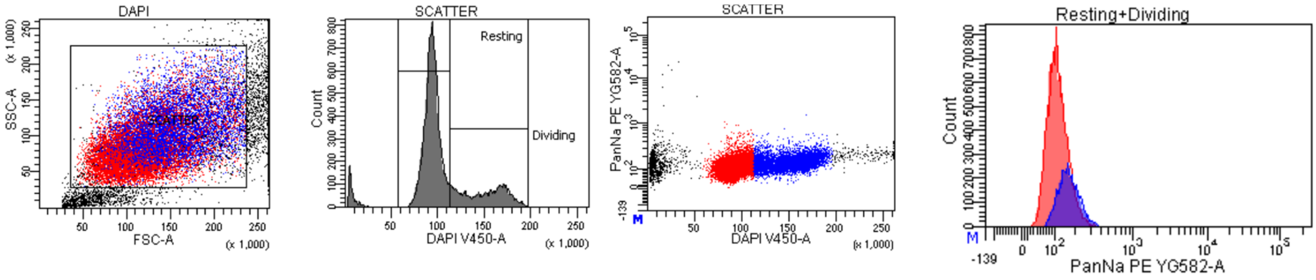

DNA label + Na,K-ATPase labeled:

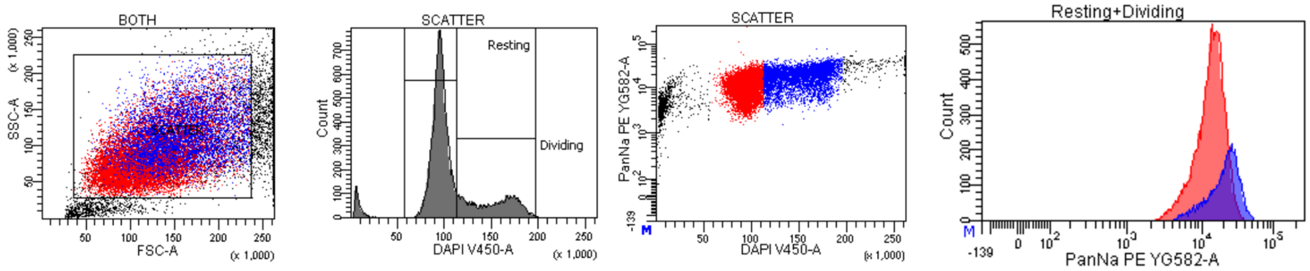

Figure S9. H28 Na,K-ATPase -RPE + DAPI Flow Cytometry Plots

MeT-5a: Na,K-ATPase -PE +

Vehicle Only:

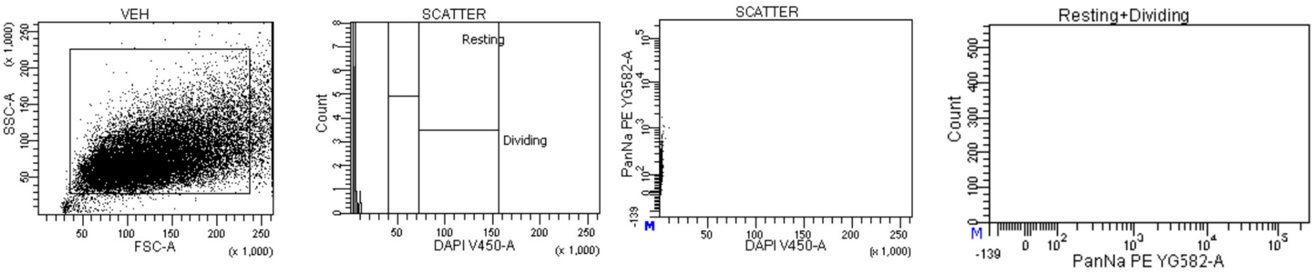

Na,K-ATPase labeled only:

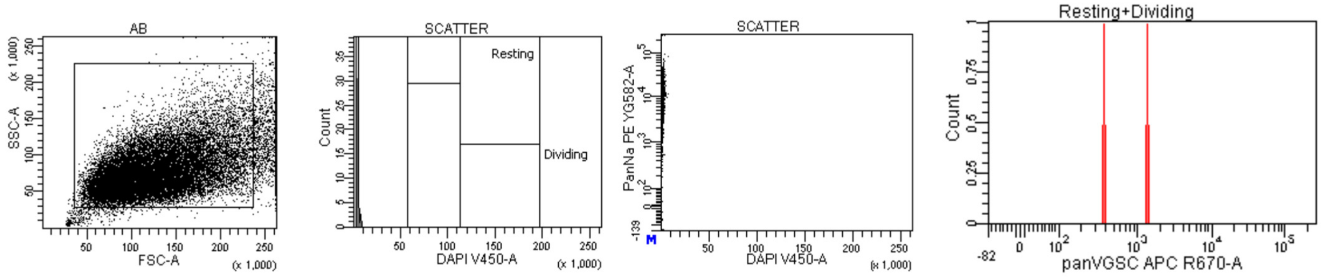

DNA label only:

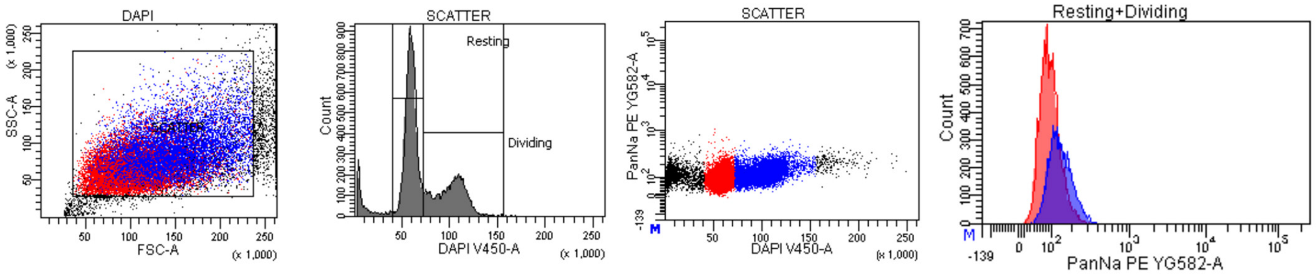

DNA label + Na,K-ATPase labeled:

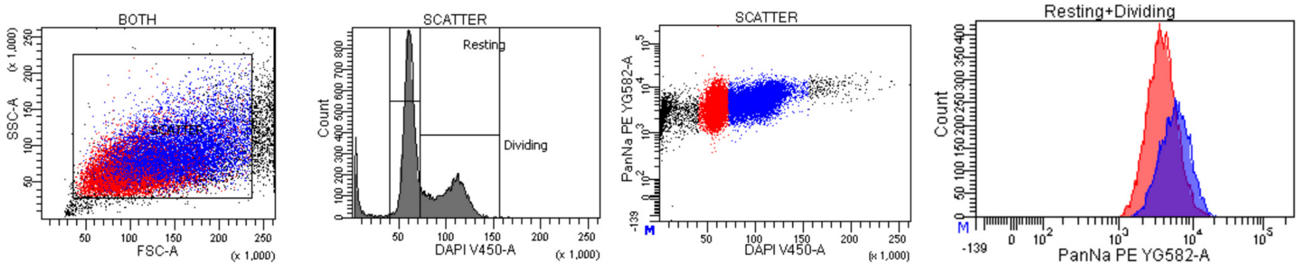

Figure S10. MeT-5a Na,K-ATPase -RPE + DAPI Flow Cytometry Plots

AB1: Na,K-ATPase-PE + DAPI

Vehicle Only:

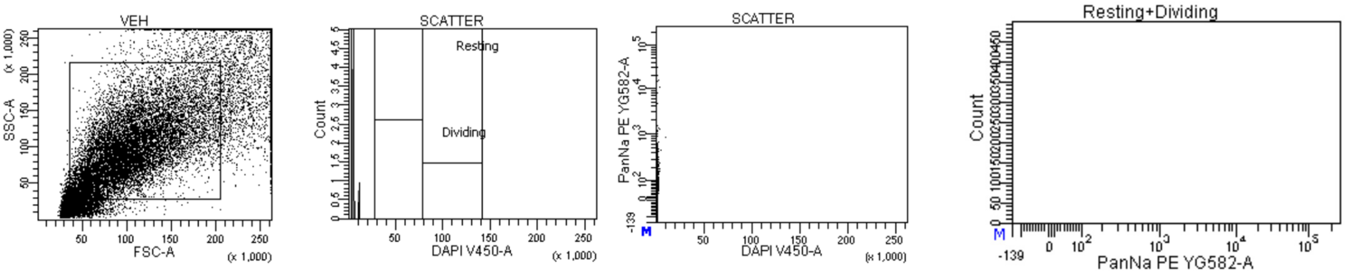

Na,K-ATPase labeled only:

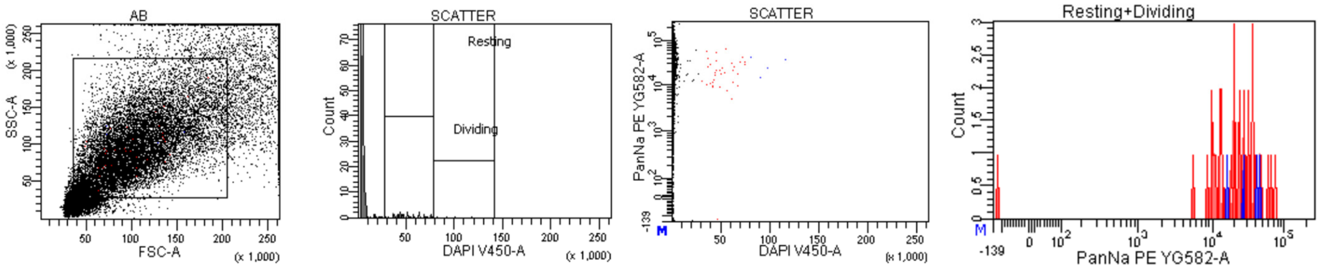

DNA label only:

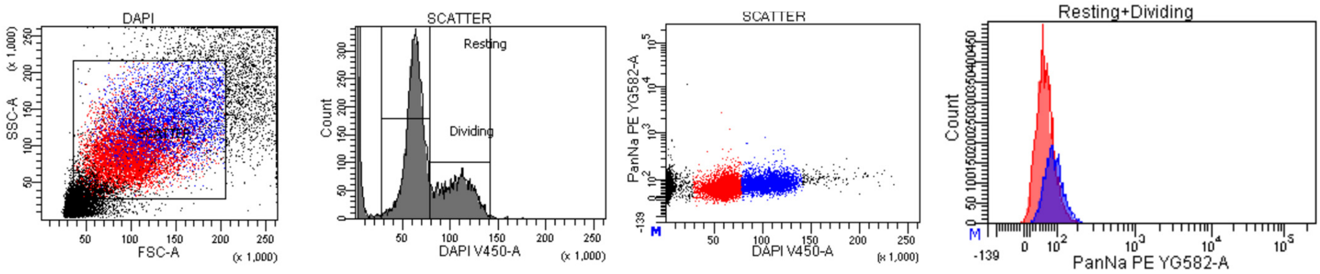

DNA label + Na,K-ATPase labeled:

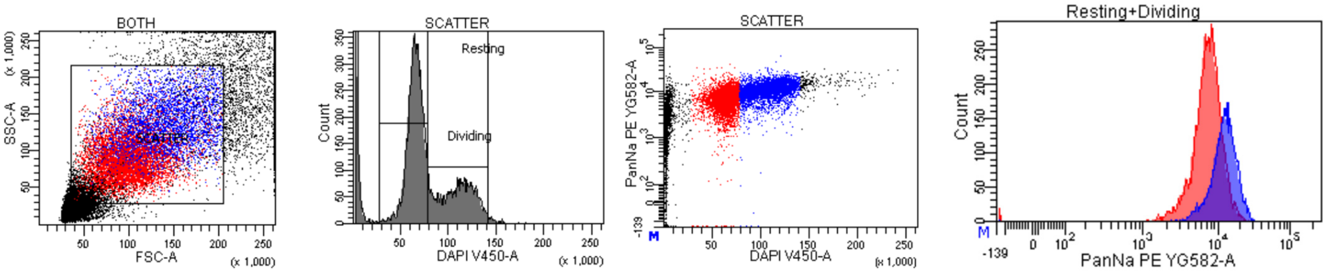

Figure S11. AB1 Na,K-ATPase -RPE + DAPI Flow Cytometry Plots

MDA-MB-231: Na,K-ATPase -PE + DAPI

Vehicle Only:

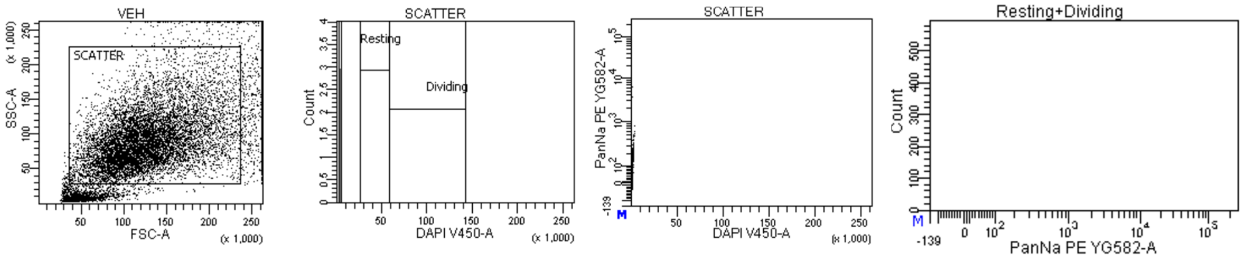

Na,K-ATPase labeled

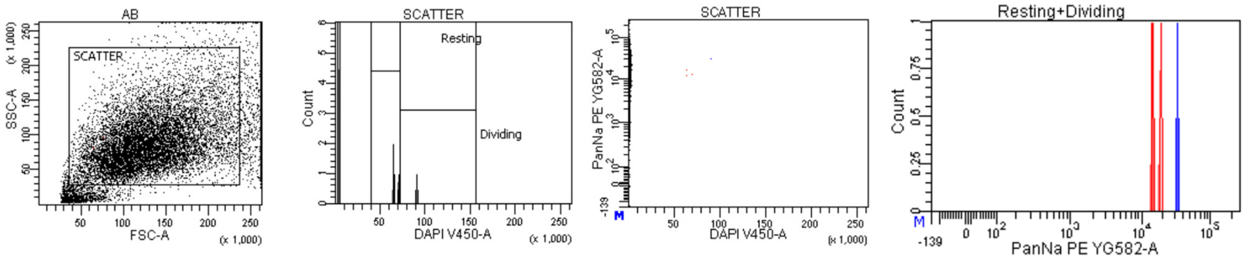

DNA label only:

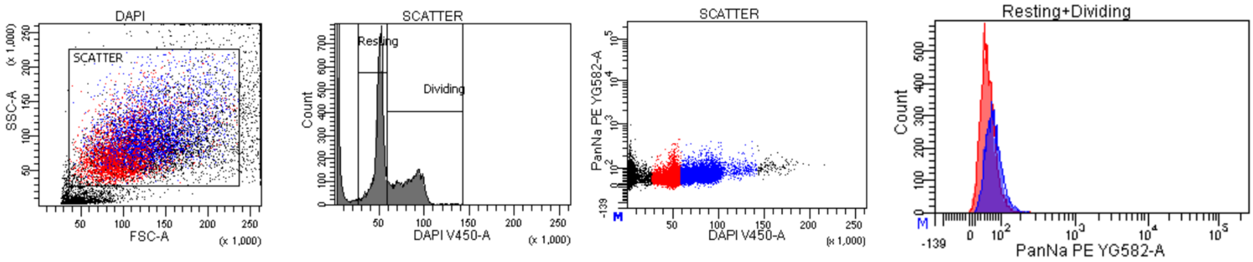

DNA label + Na,K-ATPase

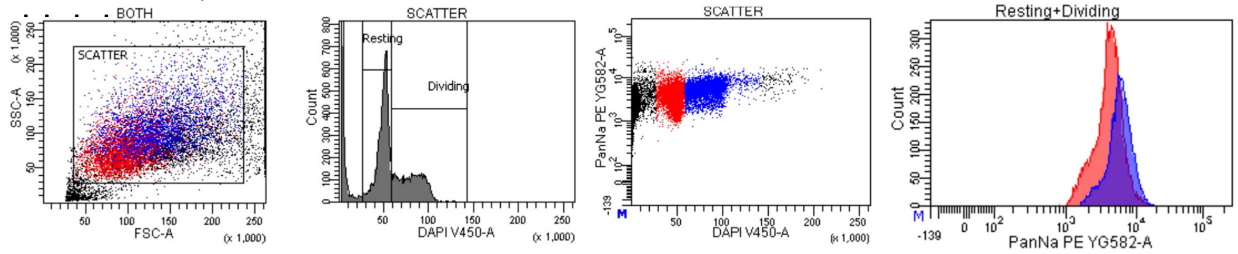

Figure S12. MDA-MB-231 Na,K-ATPase -RPE + DAPI Flow Cytometry Plots

MCF-10a: Na,K-ATPase -PE + DAPI

Vehicle Only:

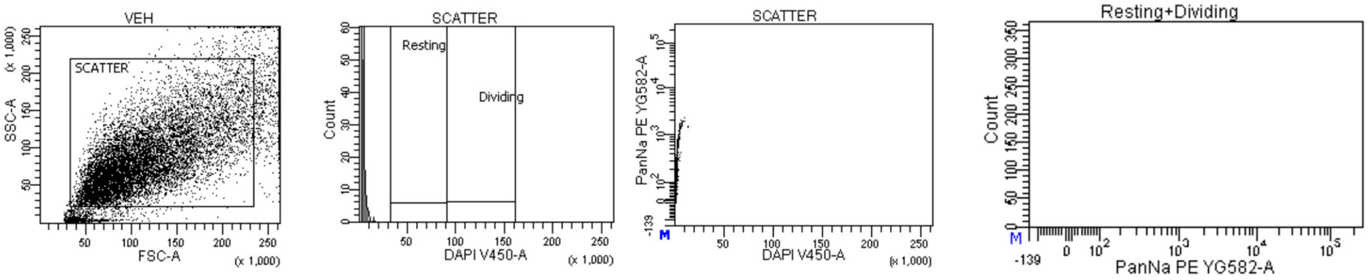

Na,K-ATPase labeled only:

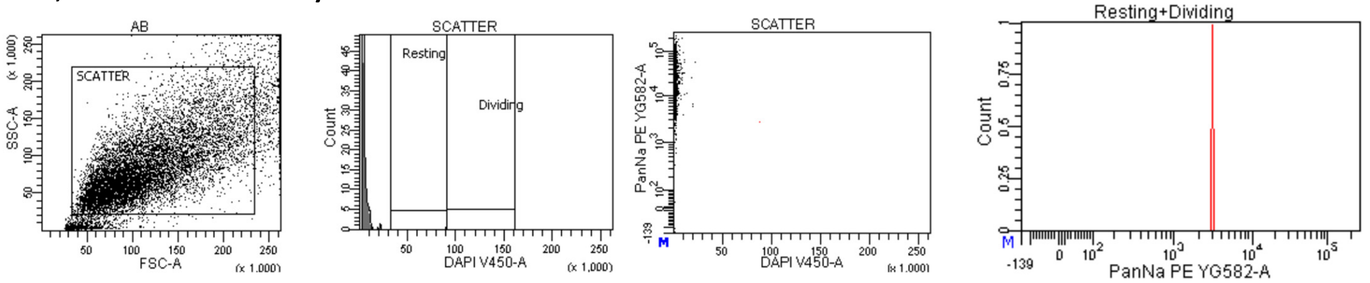

DNA label only:

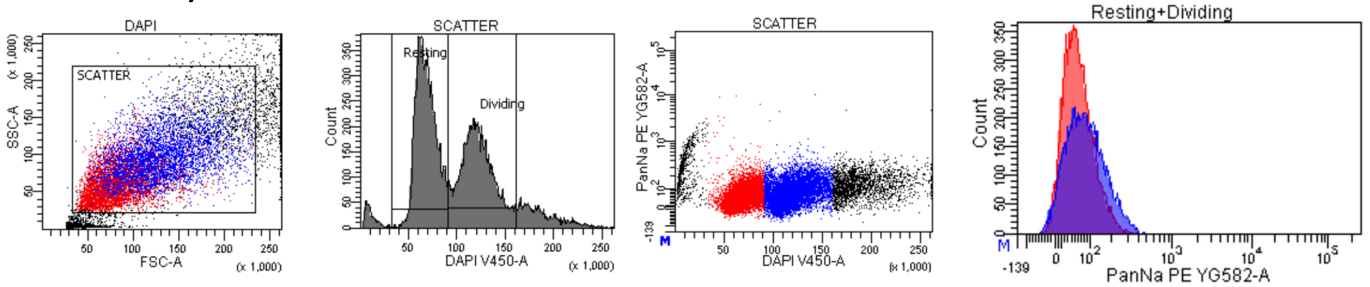

DNA label + Na,K-ATPase labeled:

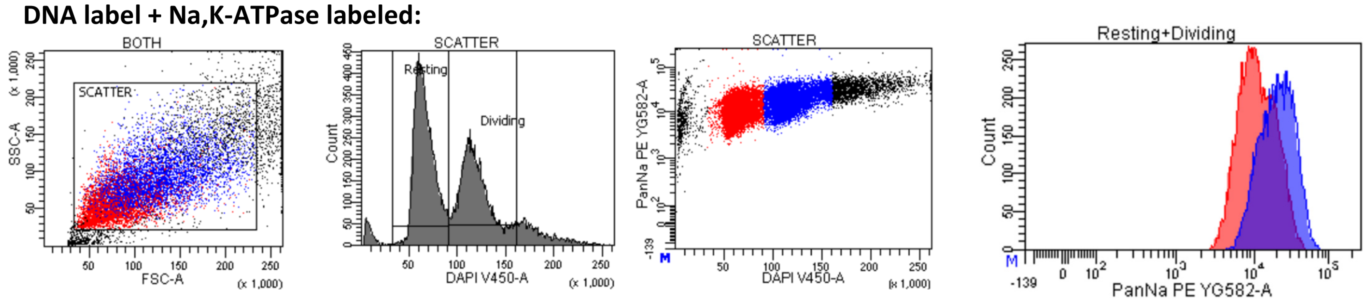

Figure S13. MCF-10a Na,K-ATPase -RPE + DAPI Flow Cytometry Plots

4T1: Na,K-ATPase -PE + DAPI

Vehicle Only:

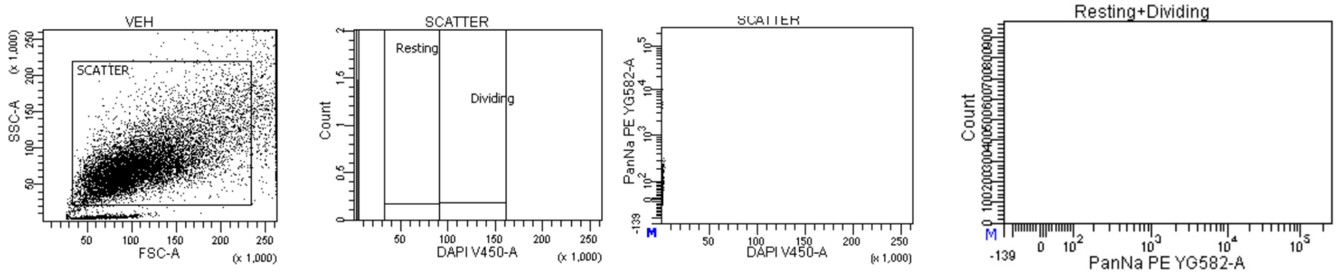

Na,K-ATPase labeled only:

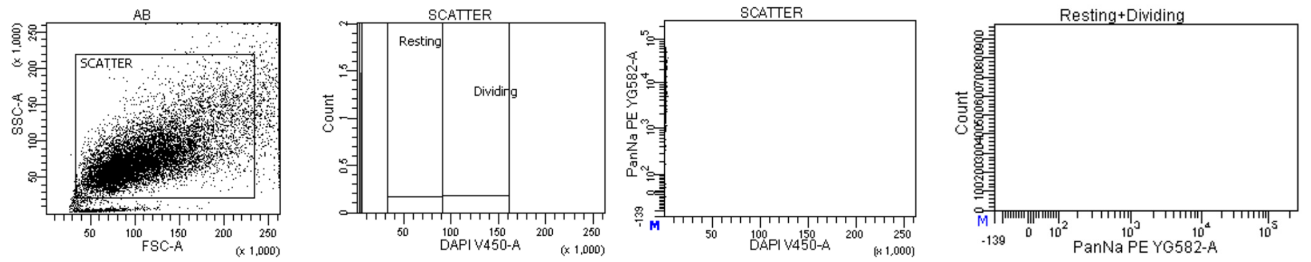

DNA label only:

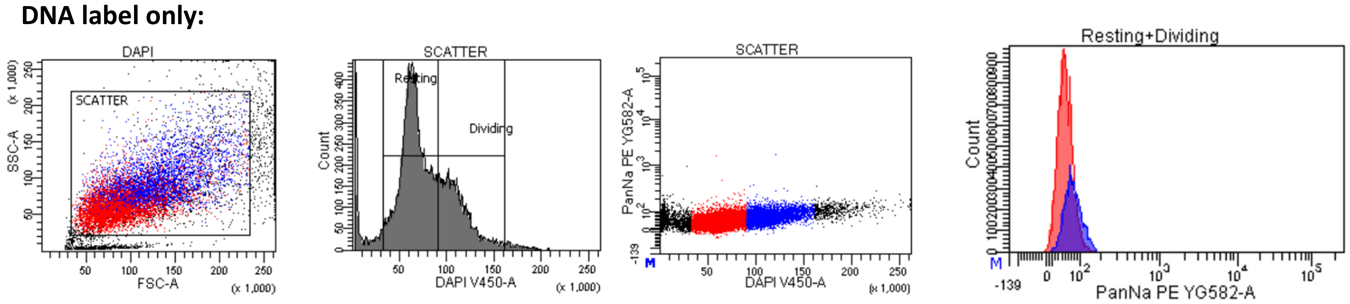

DNA label + Na,K-ATPase labeled:

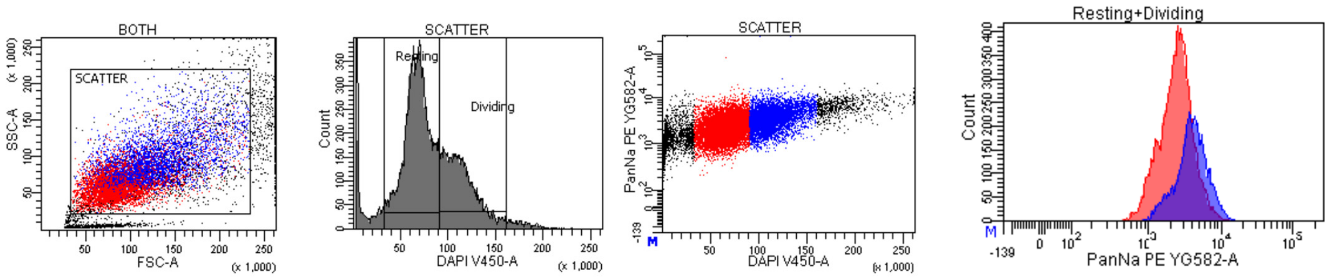

Figure S14. 4T1 Na,K-ATPase -RPE + DAPI Flow Cytometry Plots

A549: Na,K-ATPase -PE + DAPI

Vehicle Only:

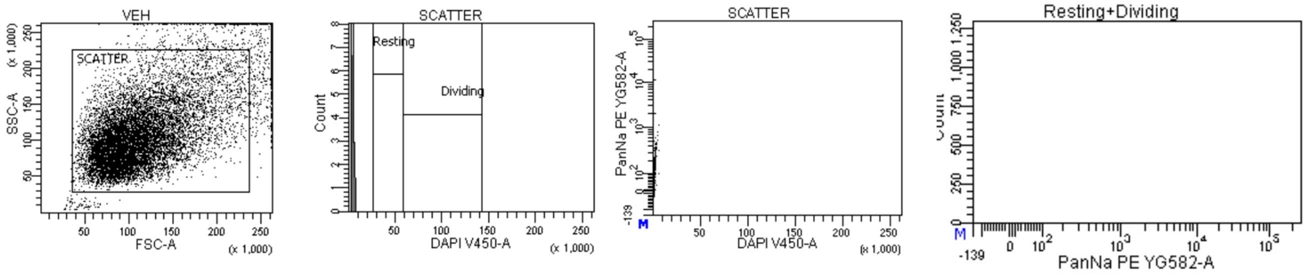

Na,K-ATPase labeled only:

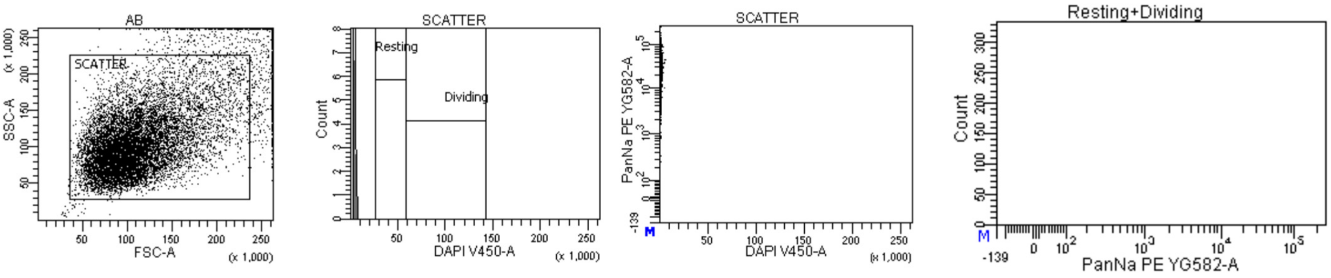

DNA label only:

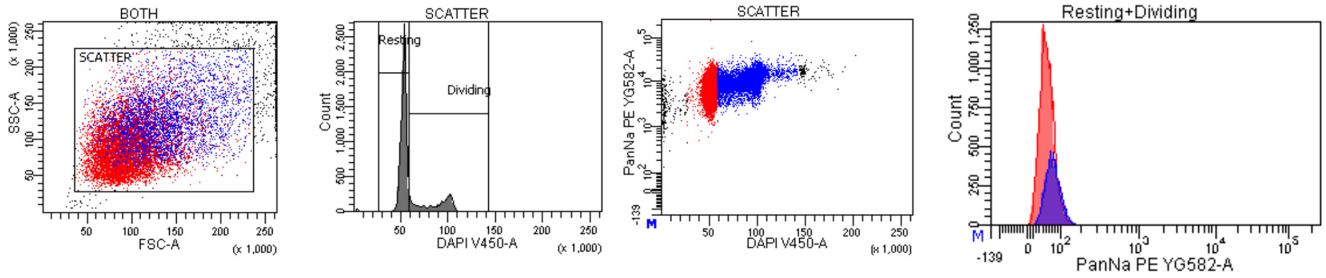

DNA label + Na,K-ATPase labeled:

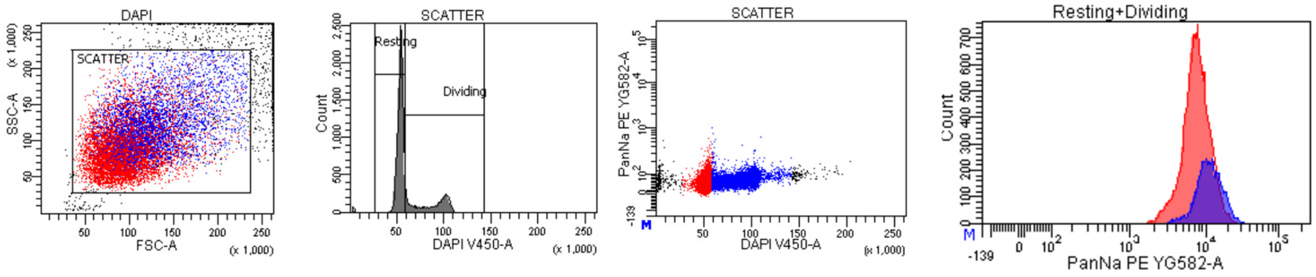

Figure S15. A549 Na,K-ATPase -RPE + DAPI Flow Cytometry Plots

MRC5: Na,K-ATPase -PE +

Vehicle Only:

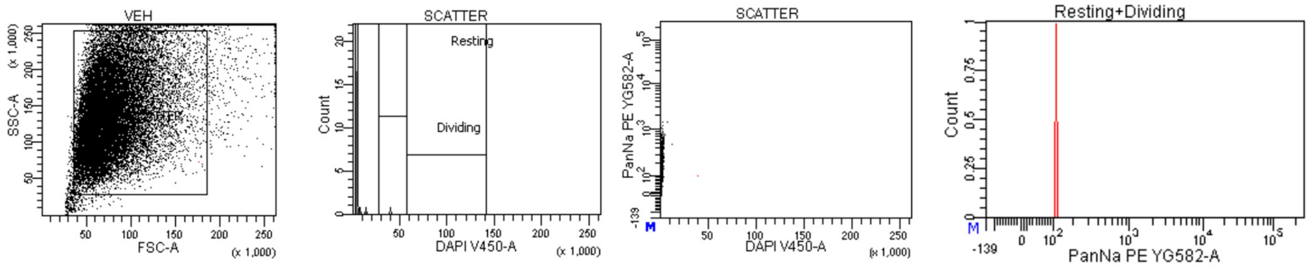

Na,K-ATPase labeled only:

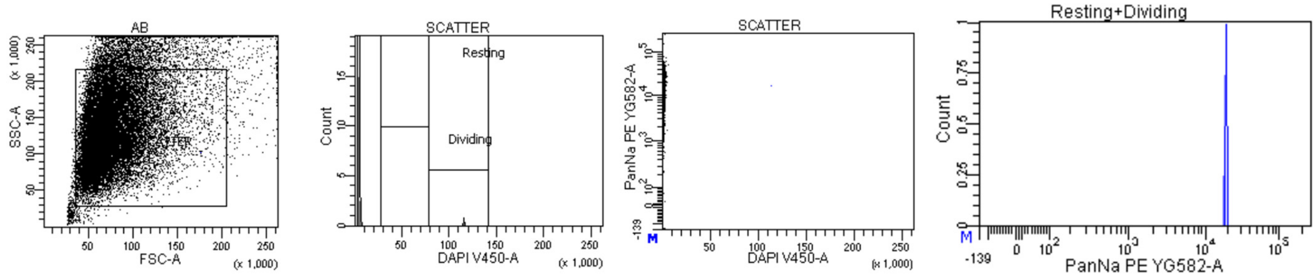

DNA label only:

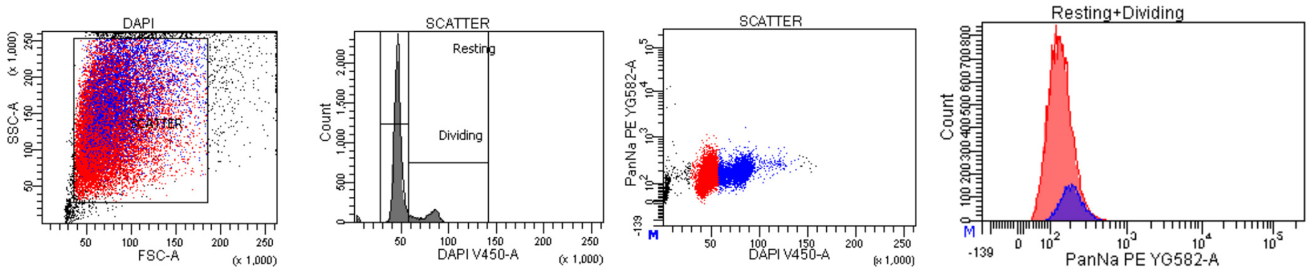

DNA label + Na,K-ATPase labeled:

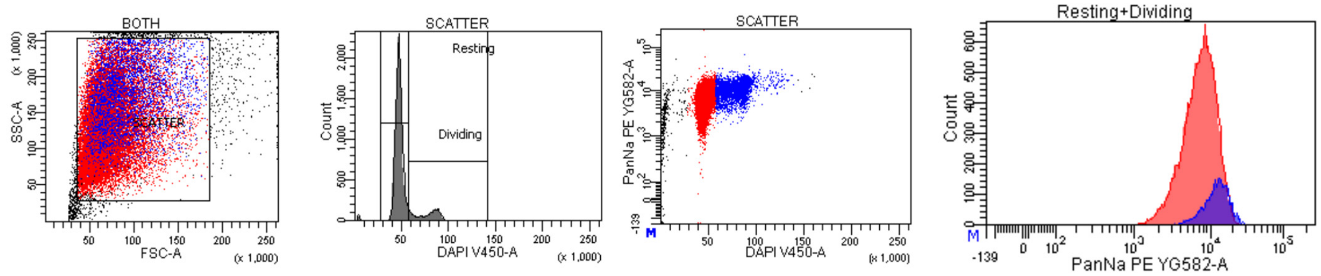

Figure S16. A549 Na,K-ATPase -RPE + DAPI Flow Cytometry Plots
